# Intrauterine hyperglycemia impairs mouse primordial germ cell development and fertility by sex-specific epigenetic reprogramming interference

**DOI:** 10.1038/s41421-025-00821-0

**Published:** 2025-09-09

**Authors:** Jiangshan Cong, Qing Li, Yangyang Li, Minghao Li, Yan Shi, Peiran Hu, Xidi Yin, Qianyun Zhang, Jianzhong Sheng, Jinsong Li, Guolian Ding, Yu Zhang, Hefeng Huang

**Affiliations:** 1 Obstetrics and Gynecology Hospital, Institute of Reproduction and Development, Fudan University, Shanghai, China; 2 Shanghai Key Laboratory of Reproduction and Development, Shanghai, China; 3 Department of Histoembryology, Genetics and Developmental Biology, Shanghai Key Laboratory of Reproductive Medicine, Shanghai Jiao Tong University School of Medicine, Shanghai, China; 4 Key Laboratory of Multi-Cell Systems, Shanghai Key Laboratory of Molecular Andrology, Center for Excellence in Molecular Cell Science, Shanghai Institute of Biochemistry and Cell Biology, Chinese Academy of Sciences, Shanghai, China; 5 Department of Obstetrics and Gynecology, Center for Reproductive Medicine, the Fourth Affiliated Hospital of School of Medicine, and International School of Medicine, International Institutes of Medicine, Zhejiang University, Yiwu, Zhejiang China; 6 Key Laboratory of Reproductive Genetics (Ministry of Education) and Department of Reproductive Endocrinology, Women’s Hospital, Zhejiang University School of Medicine, Hangzhou, Zhejiang China; 7 Institute of Medical Genetics and Development, Zhejiang University, Hangzhou, Zhejiang China

**Keywords:** Embryonic germ cells, Reprogramming, Epigenetics

## Abstract

Adverse intrauterine environments, such as hyperglycemia, impair sexual reproduction and species continuity, yet the underlying mechanisms remain poorly understood. In this study, we demonstrated that intrauterine hyperglycemia significantly disrupted primordial germ cell (PGC) development, especially in female offspring, thus reducing fertility. Using *Oct4-EGFP* transgenic mice with intrauterine hyperglycemia exposure, we revealed that hyperglycemia compromised sexually specific chromatin accessibility and DNA methylation reprogramming during PGC development. Particularly, in female PGCs, hyperglycemia leads to the aberrant retention of chromatin accessibility at pluripotency gene promoters such as *Nanog* and *Tfap2c*, inhibiting proper gene silencing and blocking the initiation of meiosis, which ultimately hinders oocyte maturation. Conversely, male PGCs exhibit less severe changes in chromatin accessibility and gene transcription. Intriguingly, the global DNA methylation reconstruction is impaired in male PGCs, particularly in key imprinted gene regions, suggesting potential developmental ramifications for later stages and even subsequent generations. Particularly, our findings indicate that intrauterine hyperglycemia adversely affects sex differentiation in PGCs by disrupting the expression of critical sex-determining transcription factors. Collectively, these findings highlight how intrauterine hyperglycemia interferes with sex-specific epigenetic reprogramming during PGC development, leading to abnormal germ cell development, reduced fertility, and adverse intergenerational effects.

## Introduction

Exposure to an adverse intrauterine environment can induce epigenetic reprogramming and alter gene expression during critical development stages, increasing the risk of chronic diseases later in life^1–3^. Intrauterine hyperglycemia (IUHG), such as gestational diabetes mellitus (GDM), the most common complication during pregnancy^4,5^, exerts profound and enduring effects on the health of offspring^6,7^. However, the molecular mechanisms underlying the adverse effects of IUHG on the offspring’s reproductive system remain largely unexplored.

The development of PGCs and their differentiation into gametes is critical for mammal reproduction. In mice, PGC development exhibits marked sex-specific differences. For example, female PGCs gradually exit the pluripotency stage and begin meiosis around embryonic day 13.5 (E13.5), while male PGCs stay quiescent in the mitotic stage until birth^8,9^. Pluripotency genes, such as *Sox2* and *Nanog*, are essential for the migration and proliferation of PGCs^10,11^. *Stra8* is a key meiosis-associated gene that is responsible for promoting the expression of meiosis-specific genes and repressing the establishment of the somatic program in PGCs^12–14^. The normal exit from the pluripotency program and entry into meiosis are indispensable for gamete production.

Epigenetic reprogramming plays important roles in gene expression, meiosis, genome integrity, and genomic imprinting during germ cell development^15^. During PGC development, the genome-wide epigenetic reprogramming occurs concurrently with the preservation of pluripotency^16^, also showing sex-specificity. For example, chromatin landscape undergoes profound reorganization during meiosis, driven by key processes such as synaptonemal complex formation and homologous recombination^17^. The distinct timing of meiotic entry between male and female germ cells may contribute to sex-specific chromatin openness dynamics. PGCs also undergo extensive global demethylation from E8.5 to E13.5 in both sexes, affecting regulatory regions^18^. After E13.5, male PGCs begin to re-establish DNA methylation, particularly on genes and regions related to spermatogenesis^19^. By contrast, female PGCs maintain low DNA methylation levels until later stages of oocyte development^18,20^. The accurate re-establishment of the epigenetic landscapes during PGC development is crucial for setting proper gene expression. Considering the key processes of PGC development within the intrauterine environment, maternal metabolic disturbances like hyperglycemia can lead to abnormal epigenetic reprogramming and gene expression in the offspring, which requires further investigation.

Streptozotocin (STZ) is used for inducing hyperglycemia with rapid but transient in vivo activity due to its short half-life, serving as a widely used model for studying hyperglycemia-induced effects^21,22^. With this model, we proved that maternal hyperglycemia reduces TET3 in oocytes, disrupting paternal genome demethylation and affecting offspring metabolism^22^. We also demonstrated that GDM can impair male testicular development and induce DNA methylation anomalies in PGCs, thereby affecting intergenerational metabolic outcomes^23,24^. Other research has shown that hyperglycemia impairs oocyte meiosis and development in mice^25^. Despite these findings, the mechanisms of how hyperglycemia affects PGC development remain unclear when offspring PGCs and gonads are directly exposed to a high-glucose environment. Moreover, how sex differences are achieved in GDM offspring remains to be further investigated. In this study, we found that IUHG alters chromatin structure in E12.5 PGCs without transcriptional changes in pluripotent transcription factors. This disruption leads to the failure to shut down the pluripotency gene regulatory network in E13.5 female PGCs, impairing the proper entry into meiosis, leading to a reduced number of fetal oocytes and a decline in fertility. By contrast, for male PGCs, IUHG disrupts DNA methylation reprogramming, ultimately resulting in a decrease in sperm concentration and a decline in fertility. Moreover, IUHG affects fetal sexual differentiation.

## Results

### IUHG impairs fetal gonadal development and fertility in mouse offspring

**Fig. 1:**
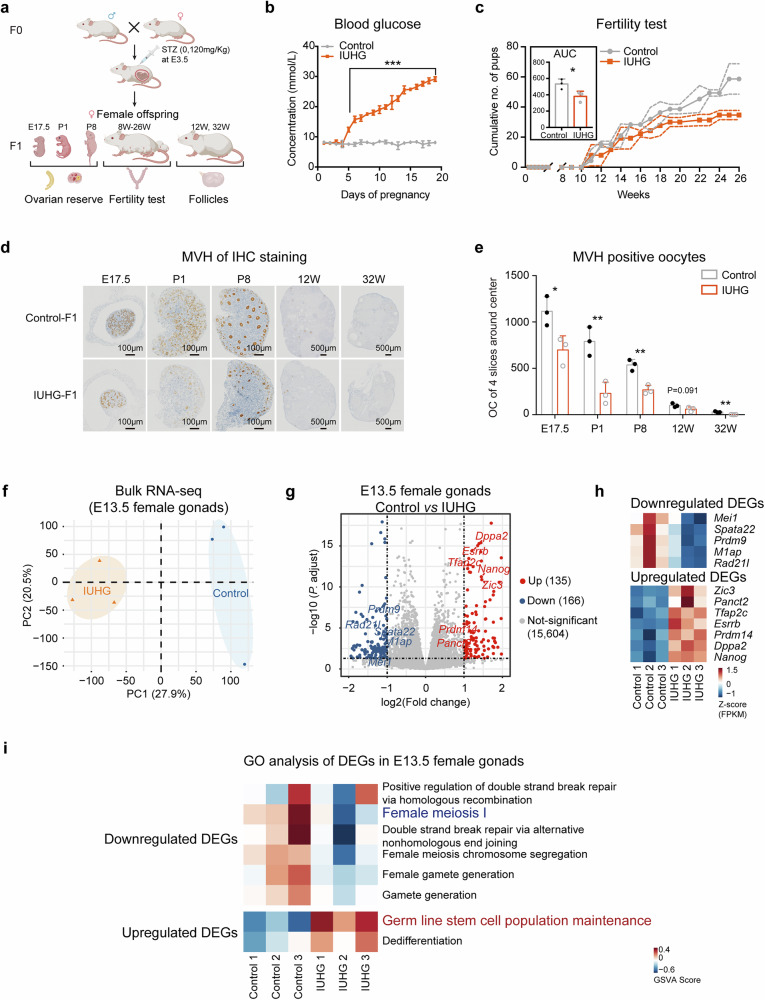
IUHG impairs the oocyte development of female offspring. **a** Diagram illustrating the construction of the IUHG model in female mice. Wild-type male and female ICR mice were mated, and the pregnant females were injected with STZ (120 mg/kg) on day 3.5 of pregnancy to induce IUHG. The female offspring were collected at E17.5, P1, P8, 12 W, and 32 W. **b** Line chart showing the blood glucose of pregnant mice between control and IUHG at E6.5, *n* = 5 dams. **c** Fertility test of female offspring, *n* = 3 pups. **d** IHC staining of MVH of the ovaries from E17.5 to 32 W, *n* = 3 pups. **e** Barplot showing the oocyte number from E17.5 to 32 W, *n* = 3 pups. **f** PCA of female gonads transcriptomes from IUHG and control group at E13.5, *n* = 3 per group. **g** Volcano plot depicting differentially expressed genes (DEGs) in IUHG female gonads at E13.5 compared to control groups (upregulated genes in red, downregulated genes in blue), *n* = 3 per group. **h** Heatmap showing expression of select DEGs in control and IUHG E13.5 female gonads. **i** Heatmap showing GO enrichment results for upregulated and downregulated DEGs in IUHG E13.5 female gonads compared to the control group. Data are presented as mean ± SD. Significance was calculated using an unpaired two-sided Student’s *t*-test (**b**, **c**, **e**); *P < 0.05, **P < 0.01, ***P < 0.001.

To investigate how IUHG affects offspring gonad development, we established a mouse model of IUHG by injection of STZ into pregnant mice to impair the pancreatic islets (Fig. 1a). Considering the pharmacokinetics of STZ and the development process of PGCs, STZ was administered on day 3.5 of pregnancy, before the emergence of embryonic PGCs, which begin to appear around E6.25^26^. The IUHG group exhibited elevated blood glucose levels after 3 days of injection, which were maintained throughout the pregnancy (Fig. 1b). After confirming the successful establishment of the model, we then examined the fertility of female offspring exposed to hyperglycemia. We observed a progressive decrease in litter size from 8 to 26 weeks, with a more significant reduction in the IUHG compared to controls (Fig. 1c). Furthermore, we examined the ovary morphology and oocytes at various development stages (E17.5, postnatal day 1 (P1), P8, 12-week-age (12 W), and 32 W) using immunohistochemistry (IHC) staining (Fig. 1d). The oocyte number in IUHG female offspring is already significantly lower than that in controls after E17.5, indicating a reduced early ovarian reserve (Fig. 1e).

To delineate how IUHG affects oocyte development, we conducted bulk RNA sequencing (bulk RNA-seq) on the E13.5 female gonads, a stage when females began to enter the meiosis stage. Principal component analysis (PCA) showed a clear separation between control and IUHG female gonads (Fig. 1f), suggesting a profound effect on the transcriptome. Moreover, in E13.5 gonads of the IUHG female offspring, 135 genes were significantly upregulated and 166 genes were significantly downregulated compared to controls (Fig. 1g). To our surprise, Gene ontology (GO) analysis revealed that upregulated genes are mainly enriched in stem cell population maintenance (e.g., *Tfap2c* and *Nanog*), while most downregulated genes are associated with meiosis (e.g., *Mei1* and *Prdm9*) in IUHG E13.5 female gonads (Fig. 1h, i and Supplementary Fig. S1). These findings suggest that IUHG primarily disrupts germ cell development in female offspring, without markedly affecting ovarian structure during fetal development. Combined with our previous results^24^, these findings indicate that IUHG impairs fertility in both sexes by affecting reproductive development as early as the PGC stage.

### IUHG impairs the PGC development of offspring, especially meiosis at E13.5 in females

**Fig. 2:**
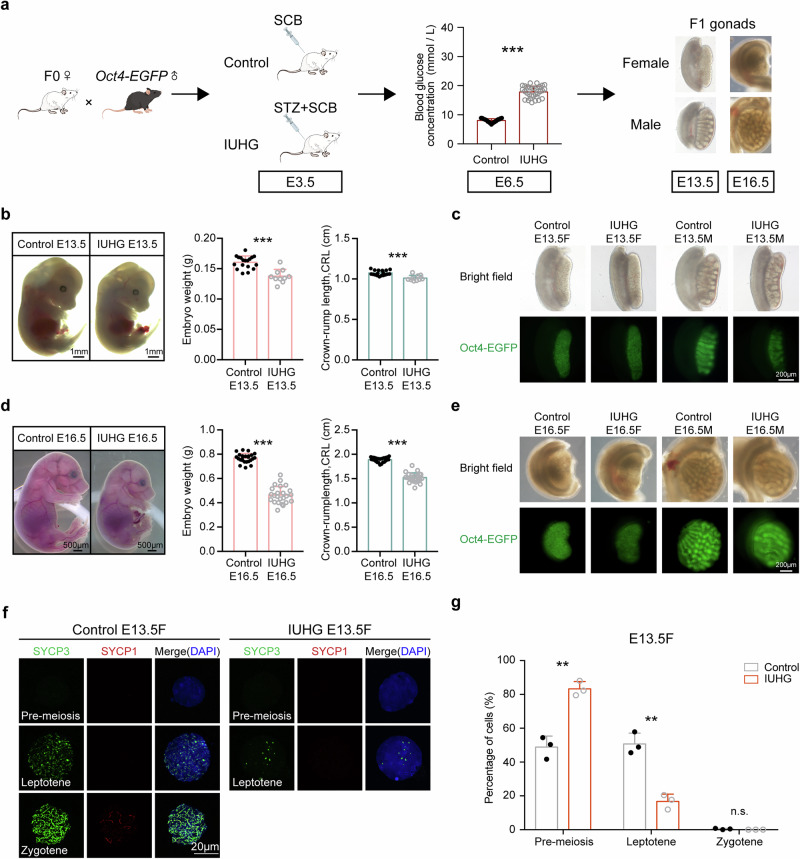
Maternal hyperglycemia affects offspring embryonic development and germ cell differentiation. **a** Schematic showing the construction of the IUHG model with *Oct4-EGFP* male mice via STZ treatment. **b** Embryonic phenotype at E13.5. Representative images and quantifications of embryo weight and crown-rump length were detected in the control (*n* = 18 litters) and IUHG (*n* = 10 litters) groups. **c** Bright-field and Oct4-EGFP fluorescence images of gonads at E13.5 in control and IUHG by fluorescence microscopy. **d** Embryonic phenotype at E16.5. Representative images and quantifications of embryo weight and crown-rump length were detected in the control (*n* = 24 litters) and IUHG (*n* = 24 litters) groups. **e** Bright-field and Oct4-EGFP fluorescence images of gonads at E16.5 in control and IUHG by fluorescence microscopy. **f** Immunofluorescence staining of meiotic markers SYCP1 (red), SYCP3 (green), and counterstained with DAPI (blue) for nuclei in chromosome spreads of the oocyte at E13.5. **g** Distribution of meiotic stages in oocytes from E13.5 fetuses in control and IUHG. A minimum of 200 cells was counted per experiment with three biological replicates conducted. Data are presented as mean ± SD. Significance was calculated using an unpaired two-sided Student’s *t*-test (**a**, **b**, **d**, **g**); **P < 0.01, ***P < 0.001, n.s. not significant.

Based on our bulk RNA-seq data, germ cell development was severely disrupted. To further explore the molecular mechanisms of IUHG on PGCs, we conducted experiments on the *Oct4-EGFP* transgenic model, which specifically labels PGCs in the gonads^27^. The IUHG group exhibited glucose levels exceeding 14 mmol/L following STZ injection at E6.5, establishing a typical IUHG environment (Fig. 2a). Consistently, the effects of IUHG on F1 generation oocytes, including growing oocytes from P1, P8, and 12-week-old ovaries, were also observed in the transgenic mouse strain (Supplementary Fig. S2a, b). At P1 and P8, when the primordial follicle pool was established, ovaries in the IUHG contained fewer oocytes than controls, indicating a reduced initial ovarian reserve in IUHG females (Supplementary Fig. S2b). Additionally, ovary weight was significantly lower in IUHG females at 12 weeks, accompanied by an increase in primordial follicles and a reduction in growing follicles (Supplementary Fig. S2a, b). These results confirmed that the effect of hyperglycemia on female fetuses was consistent in different mouse strains. We also found that male offspring exposed to IUHG exhibited a significant decline in fertility (Supplementary Fig. S2c). Although sperm motility was preserved, sperm concentration declined significantly, indicating impaired male germ cell development under IUHG (Supplementary Fig. S2d–f), consistent with our previous study^23^. By contrast, bulk RNA-seq revealed disrupted pluripotency maintenance and impaired meiotic entry in female PGCs (Fig. 1i). Since female germ cells begin meiosis in utero around E13.5, while male germ cells initiate meiosis postnatally, this may underlie the sex-specific effects observed.

Then we focused on the embryo and germ cell development from E12.5 to E16.5. Interestingly, IUHG fetuses showed a clear developmental delay, with reduced embryo weight and crown-rump length at E13.5 and E16.5 (Fig. 2b, d). Using Oct4-EGFP reporter, we visualized PGCs and gonadal morphology in both control and IUHG embryos at E13.5 and E16.5 (Fig. 2c, e), where growth deficits persisted. Thus, to further determine whether the meiosis process is indeed disrupted in the fetal ovaries of the IUHG, we examined chromosome behavior at E13.5, when the female germ cell is reported to initiate meiosis and form synaptonemal complex^26^. Compared to controls, most IUHG oocytes had not entered prophase I at this stage (Fig. 2f and Supplementary Fig. S3a). Additionally, the proportion of oocytes in the leptotene stage was significantly lower in IUHG fetuses (Fig. 2g), potentially leading to reduced oocyte numbers. These results suggest that IUHG impairs fetal PGC development in both sexes and specifically disrupts meiotic initiation in female germ cells.

To further elucidate the cause of reduced germ cell numbers observed in E13.5 IUHG embryos, we analyzed proliferation and apoptosis dynamics from E12.5 to E13.5. Ki67 staining revealed significantly reduced proliferation in both female and male PGCs at E12.5, which persisted through E13.5 under hyperglycemic conditions (Supplementary Fig. S3b), indicating that IUHG impairs germ cell proliferation early on. Cleaved caspase-3 staining showed no significant increase in apoptosis at E12.5; however, by E13.5, apoptosis was markedly increased in female PGCs, but not males (Supplementary Fig. S3c), suggesting a sex-specific vulnerability. These results indicate that IUHG suppresses early PGC proliferation and induces female-specific apoptosis, potentially contributing to the delayed meiotic initiation observed in E13.5 IUHG oocytes.

### Female PGCs exposed to IUHG exhibit delayed pluripotency exit and meiosis initiation

**Fig. 3:**
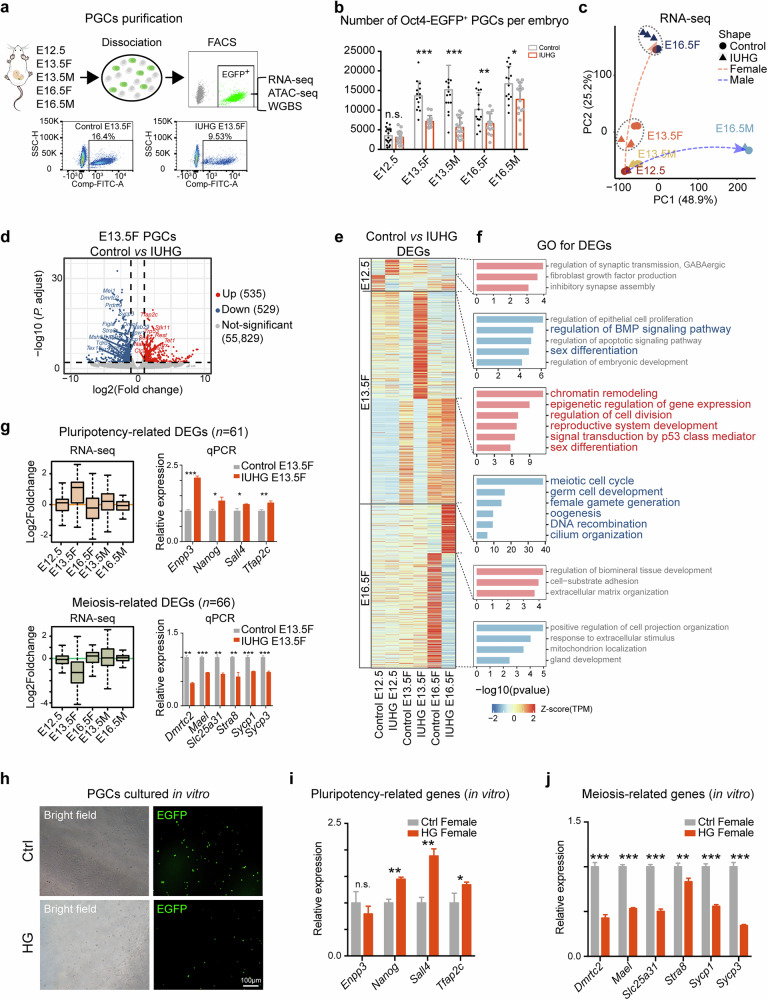
Impact of maternal hyperglycemia on fetal PGCs at E12.5 to E16.5 development stage by RNA-seq. **a** Flowchart overview of fetal PGCs isolation from E12.5 to E16.5. **b** Average number of Oct4-EGFP positive cells in whole gonads at E12.5, E13.5, and E16.5 between the control and IUHG, comprising 10–20 pairs of gonads per group. **c** PCA analysis of the developmental trajectory of fetal PGCs in control and IUHG samples based on RNA-seq data. **d** Volcano plot depicting DEGs in IUHG female PGCs at E13.5 compared to the control group (upregulated genes in red, downregulated genes in blue). **e** Heatmap showing expression of upregulated and downregulated genes in control and IUHG at E12.5, E13.5 female (E13.5 F), and E16.5 F PGCs. **f** GO analysis of DEGs identifies biological processes affected by IUHG at different development stages. **g** Boxplots of RNA-seq data and qPCR validation for pluripotency- and meiosis-related DEGs in IUHG E13.5 F PGCs compared to controls. **h** In vitro culture of E12.5 female PGCs for 24 h. **i**, **j** qPCR validation of pluripotency-related (**i**) and meiosis-related (**j**) genes in HG-treated female PGCs in vitro compared to controls. All error bars represent the mean ± SD of three biological replicates, each derived from PGCs collected from 6–10 pairs of gonads per group. Significance was calculated using an unpaired two-sided Student’s *t*-test (**b**, **g**, **i**, **j**); *P < 0.05, **P < 0.01, ***P < 0.001, n.s., not significant.

To further investigate the molecular mechanism of IUHG on PGCs, we collected the Oct4-EGFP positive PGCs from E12.5, E13.5, and E16.5 gonads and performed RNA-seq (Smart-seq2)^28^ (Fig. 3a). Quantification of Oct4-EGFP positive PGCs showed a significant reduction in germ cell numbers in IUHG gonads compared to controls (Fig. 3b). To exclude potential off-target effects of STZ itself, we included a group of STZ-injected pregnant mice without elevated blood glucose (NonHG group), in which no significant differences in Oct4-EGFP positive PGC numbers (Supplementary Fig. S4). Next, PCA analysis of RNA-seq data confirmed distinct development trajectories for female and male PGCs (Fig. 3c). Additionally, we found that transcription of control and IUHG PGCs did not show significant change at E12.5 (Fig. 3c), consistent with the comparable PGC number at E12.5 (Fig. 3b). At E13.5, female PGCs exhibited significant transcriptional changes (Fig. 3c, orange), while male PGCs were less affected, even at E16.5. The transcriptional profile in female PGCs gradually recovered by E16.5 (Fig. 3c and Supplementary Fig. S5a, b).

Since the earliest and most obvious transcriptional differences were observed at E13.5 in female PGCs, we further conducted differential gene expression analysis at this stage, identifying 529 downregulated and 535 upregulated genes (Fig. 3d and Supplementary Fig. S5c). GO analysis revealed that upregulated genes were associated with chromatin remodeling (e.g., *Rest*, *Atrx*, and *Ctcf*), epigenetic regulation of gene expression (e.g., *Tet1*, *Tfap2c*), reproductive system development (e.g., *Six4*), signal transduction by p53 class mediator (e.g., *Trp53*, *Stk11*), regulation of cell division (e.g., *Nanog*), and sex differentiation (e.g., *Sox2*). By contrast, the majority of downregulated genes were enriched in processes such as meiotic cell cycle regulation (e.g., *Dmrtc2*, *Stra8*, *Sycp1*, *Sycp3*, and *Tex15*), germ cell development (e.g., *Mei1*, *Tdrd1*), female gamete generation (e.g., *Prdm9*, *Terb2*, and *Msh5*), oogenesis (e.g., *Figla*, *Dmc1*), DNA recombination (e.g., *Syce3*, *Zcwpw1*), cilium organization (e.g., *Evi5l*, *Rab29*, and *Ift122*) in E13.5 IUHG fetal oocytes (Fig. 3e, f and Supplementary Fig. S6a, b). We also examined DEGs in E13.5 IUHG male PGCs, identifying 603 downregulated and 559 upregulated genes, with significant enrichment in the *Wnt* signaling pathway, cilium movement, and germ cell development pathways (Supplementary Fig. S6c–f). Therefore, the changes in the expression of these DEGs may directly influence the spermatogenesis process, ultimately leading to reduced sperm concentration (Supplementary Fig. S2f). This suggests that the impact of IUHG on male offspring begins during the embryonic stage. Additionally, we observed that at E12.5, downregulated DEGs were associated with the “BMP signaling pathway” and “sex differentiation”, whereas at E16.5, both female and male, they were primarily related to “developmental processes” (Fig. 3e, f and Supplementary Fig. S6c, d). These results highlight that hyperglycemia exerts sexual- and stage-specific impacts during PGC development.

Of note, a previous study revealed that female PGCs must normally exit pluripotency to successfully enter meiosis at E13.5^26^. Building on this, we analyzed two gene clusters among our DEGs: 61 pluripotency-related genes and 66 meiosis-related genes (Supplementary Fig. S6g, h and Tables S1, S2), including key pluripotency markers, such as *Enpp3*, *Nanog*, *Sall4*, and *Tfap2c*, and meiotic markers, such as *Dmrtc2*, *Mael*, *Slc25a31*, *Stra8*, *Sycp1*, and *Sycp3* (Supplementary Fig. S7a, b). These findings were further validated by qPCR, which aligned with the RNA-seq results (Fig. 3g, right). Immunofluorescence staining at E13.5 revealed an increase in SALL4 and a reduction in STRA8 signals in IUHG female PGCs (Supplementary Fig. S7c, d). Notably, the up/down-regulation of pluripotency genes and meiosis genes in E13.5 female PGCs was largely recovered when developed into E16.5 (Fig. 3g, left), indicating that part of female germ cells could successfully exit the pluripotent state and initiate meiosis. PGCs that could not overcome the developmental barrier may enter the process of apoptosis and eventually die as the p53 signal transduction was induced in E13.5 female PGCs (Fig. 3e, f), which may account for the cell number reduction of E13.5 female PGCs (Fig. 3b). By contrast, IUHG male PGCs showed minimal changes in pluripotency and meiosis-related gene expression at both E13.5 and E16.5 (Fig. 3g and Supplementary Fig. S8), likely because male meiosis begins postnatally. Notably, *Stra8*, a key regulator of meiosis^12,14^, is downregulated in IUHG female PGCs but upregulated in male PGCs at E13.5 (Fig. 3g and Supplementary Fig. S8d), highlighting a sex-specific response. In females, reduced *Stra8* expression may reflect delayed or disrupted meiotic entry, while its premature upregulation in males may indicate altered preparatory pathways for spermatogenesis.

To investigate whether the gene expression changes in PGCs were directly caused by hyperglycemia, we collected PGCs from E12.5 and cultured them under high glucose for 24 h (Supplementary Fig. S9a). The results showed weakened fluorescence signals and a reduced number of PGCs in both female and male cultures after 24-h high glucose treatment, which was consistent with the in vivo findings (Fig. 3h and Supplementary Fig. S9b, c). These PGCs were then collected for qPCR analysis. Pluripotency markers (such as *Nanog*, *Sall4*, and *Tfap2c*) were significantly upregulated in HG-treated female PGCs, while meiotic markers (such as *Dmrtc2*, *Mael*, *Slc25a31*, *Stra8*, *Sycp1*, and *Sycp3*) were significantly downregulated in HG-treated female PGCs (Fig. 3i, j). In HG-treated male PGCs, the expression of pluripotency genes was upregulated compared to the control group, and meiosis genes were downregulated, except for *Stra8*, which showed upregulated expression (Supplementary Fig. S9d, e). These results again aligned with the in vivo experiments. Together, these data suggest that IUHG leads to abnormal maintenance of the pluripotency network and thus blocks the entry of meiosis in female but not male PGCs. The sexually dimorphic effects on PGC development may primarily be attributed to differences in their developmental progress.

### IUHG alters the chromatin accessibility of female PGCs

**Fig. 4:**
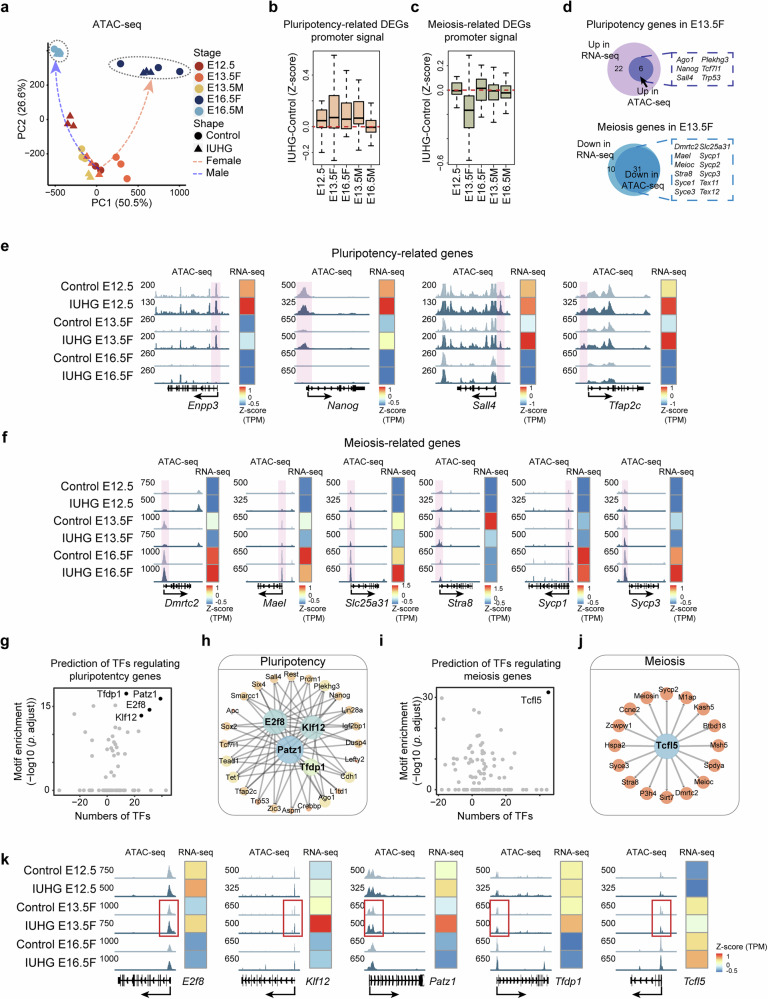
Chromatin accessibility defects lead to the abnormal transition from pluripotency to meiosis in fetal PGCs exposed to IUHG. **a** PCA analysis of the developmental trajectory of fetal PGCs based on ATAC-seq data. **b**, **c** Boxplots of promoter signal accessibility for pluripotency (**b**) and meiosis-related (**c**) DEGs in IUHG conditions compared to control groups. **d** Venn diagrams demonstrate the overlap of pluripotency and meiosis genes (at E13.5 F), which have synergistic changes of RNA-seq and nearby ATAC-seq chromatin changes. **e** Genome browser track view showing the ATAC signal enrichment at core pluripotency TF binding sites around the *Enpp3*, *Nanog*, *Sall4*, and *Tfap2c* loci in female PGCs at E12.5, E13.5, and E16.5 in control and IUHG. The heatmap shows the expression of those genes. **f** Genome browser view showing the ATAC-seq signal enrichment at core meiosis TF binding sites around the *Dmrtc2*, *Mael*, *Slc25a31*, *Stra8*, *Sycp1*, and *Sycp3* loci in female PGCs at E12.5, E13.5, and E16.5 in control and IUHG. The heatmap shows the expression of those genes. **g**–**j** Prediction of TF and regulatory networks that regulate pluripotency (**g**, **h**) and meiosis-associated (**i**, **j**) genes. The horizontal axis represents the difference in the number of genes of interest regulated by TFs under control and IUHG conditions, and the vertical axis represents the enrichment of TF motifs in DAR near the genes of interest. **k** Genome browser view showing the dynamic chromatin accessibility changes at TF binding sites around the *E2f8*, *Klf12*, *Patz1*, *Tfdp1*, and *Tclf5* loci in female PGCs at E12.5, E13.5, and E16.5 in control and IUHG. The heatmap shows the expression of those genes.

How are those genes involved in stem cell maintenance and abnormal meiosis initiation affected under hyperglycemia? Chromatin accessibility plays an essential role in gene regulation and cell differentiation. During PGC development, extensive chromatin remodeling occurs to ensure accurate cell fate determination, and any alterations to this process can impair the proper expression of key genes^29^. GO analysis also showed a significant correlation with “chromatin remodeling” in E13.5 IUHG female upregulated genes (Fig. 3f and Supplementary Fig. S6b). Thus, we collected Oct4-EGFP-positive germ cells from E12.5, E13.5, and E16.5 PGCs for Assay for Transposase-Accessible Chromatin using sequencing (ATAC-seq). The clustering results showed high reproducibility across three biological replicates for each group, with a more significant difference between E12.5 and E13.5 female PGCs (Supplementary Fig. S10). Interestingly, in contrast to the RNA-seq result, the PCA analysis of ATAC-seq revealed substantial differences in chromatin structure as early as E12.5 in PGCs (Fig. 4a). These differences remained consistently pronounced in female PGCs from E13.5 to E16.5, while changes in chromatin structure in male PGCs were relatively minor over the same period (Fig. 4a). At E12.5, the differentially accessible regions (DARs) began to change dramatically in both enhancer and promoter regions in IUHG (Supplementary Fig. S11a, b). GREAT (Genomic Regions Enrichment of Annotations Tool) GO analysis of closing enhancer DARs in IUHG E12.5 PGCs, enriched in blastocyst development and regulation of histone methylation. While in the promoter regions, the enriched functions included cell cycle DNA replication, protein localization to chromosome, mitotic sister chromatid segregation, chromatin maintenance, and spindle checkpoint (Supplementary Fig. S11c, d). Although numerous opening DARs were observed in IUHG E16.5 female PGCs, they were not clustered into relevant terms (Supplementary Fig. S11a, b). More DEGs were found near opening DARs in IUHG PGCs compared to controls, which may reflect the cumulative effect of chromatin remodeling during PGC development (Supplementary Fig. S11e). Consistent with RNA-seq results, female PGCs exhibited more significant chromatin changes in response to the high-glucose environment than male PGCs. Importantly, chromatin structure in IUHG E12.5 PGCs changed earlier than gene expression alterations, indicating its regulatory role in IUHG-dependent developmental defects.

Since chromatin openness is reported to be closely related to gene expression, we examined the chromatin accessibility of the promoter regions of important pluripotency and meiosis-related DEGs. Surprisingly, we observed that the ATAC-seq promoter signals of pluripotency-related DEGs were significantly upregulated in IUHG female PGCs as early as E12.5, while their expression did not show significant changes (Figs. 3g, 4b). Then it became the most pronounced increase occurring at E13.5 (Fig. 4b). By contrast, the meiosis-related DEGs did not show a noticeable increase in chromatin opening at E12.5 (Fig. 4c), as the exit of pluripotency might be the prerequisite of meiosis initiation^30^. To be noticed, a previous study revealed that DNA methylation is pivotal for the exit of the pluripotency program in male germ cell development^31^. However, according to our whole-genome bisulfite sequencing (WGBS) data (discussed later), it did not show a clear decrease in pluripotency-related DEGs (Supplementary Fig. S16d), suggesting that DNA methylation alone may not be the principal mechanism driving the aberrant exit from pluripotency in female PGCs. This may be because, unlike male germ cells, female PGCs do not start DNA methylation reconstruction at this developmental period^18^. In sum, these results further support that chromatin accessibility reprogramming defects, but not DNA methylation, play an essential role in regulating aberrant pluripotency maintenance and meiosis initiation.

Combining RNA-seq and ATAC-seq provides a deeper understanding of gene regulation by correlating gene expression with chromatin state. Both RNA-seq and ATAC-seq show significant overlap in the genes identified in E13.5 female PGCs (Fig. 4d). Specifically, the promoter regions of pluripotency markers such as *Enpp3*, *Nanog*, *Sall4*, and *Tfap2c* exhibited high chromatin accessibility in IUHG female E13.5 PGCs, while the chromatin of meiosis-related genes, including *Dmrtc2*, *Mael*, *Slc25a31*, *Stra8*, *Sycp1*, and *Sycp3*, showed decreased accessibility in IUHG female PGCs at E13.5 (Fig. 4e, f). These changes were not observed in IUHG male PGCs at E13.5 (Supplementary Fig. S12). These findings highlight a temporal dysregulation in the epigenetic regulation of pluripotency, potentially contributing to disrupted meiotic progression.

Analysis Algorithm for Networks Specified by Enhancers (ANANSE) and Analysis of Motif Enrichment (AME) were integrated to predict the transcriptional regulators involved in pluripotency and meiotic processes. The analysis focused on the enrichment of transcription factor (TF) motifs in peaks near the relevant gene sets, along with the number of genes regulated by pluripotency and meiotic markers. Based on these predictions, candidate pluripotency TFs, such as E2f8, Klf12, Patz1, and Tfdp1, were predicted as potential regulators of pluripotency in IUHG female PGCs (Fig. 4g, h and Supplementary Table S3). Notably, *Patz1* and *Tfdp1* knockout mice exhibit reproductive-related phenotypes such as delayed embryo development and reduced fertility^32,33^. Meanwhile, the candidate meiosis TF, Tcfl5, may play a role in the process of meiosis at IUHG female PGCs (Fig. 4i, j). It has been shown that *Tcfl5* deficiency leads to impaired meiosis, resulting in defects in sperm development and fertility^34^. The TF driving different state transitions were sorted according to influence scores generated by ANANSE, and the TFs with top influence scores were screened. It was found that some driving TFs for state transitions at E13.5 F were consistent with our previous predictions, which enhanced the reliability of our conclusions (Supplementary Fig. S13). To further investigate the relationship between pluripotency or meiosis genes and the identified transcriptional regulators in chromatin-accessible regions, we integrated gene expression and chromatin accessibility profiles during the development of female PGCs from E12.5 to E16.5 (Fig. 4k). This analysis suggests that the disruption of meiosis initiation in IUHG female E13.5 PGCs, likely due to the delayed removal of pluripotency TFs occupancy. These findings demonstrate that key characteristics of PGCs in the hyperglycemic environment during development are directly influenced by the reconfiguration of chromatin accessibility.

To explore the mechanisms of epigenetic dysregulation in IUHG-exposed germ cells, we examined histone-modifying enzymes and metabolite-linked cofactors. In female PGCs, we observed marked upregulation of *Prdm9* and *Zcwpw1/2*, key regulators of H3K4me3 deposition during meiotic chromatin remodeling, alongside elevated *Ep300*, a major histone acetyltransferase (Supplementary Fig. S14a). Consistent with gene expression, H3K4me3 levels were significantly reduced. H3K27ac showed no significant increase, but we could not exclude the role of other histone acetylation in this process (Supplementary Fig. S14b, c). Except for the expression of enzymes, metabolic intermediates also play important roles in modulating epigenetic landscapes. Given the well-known link between α-ketoglutarate (α-KG) and demethylase activity^35^, we quantified α-KG in E13.5 PGCs. We observed a significant elevation in α-KG in the hyperglycemia-exposed female group (Supplementary Fig. S15a), consistent with reduced H3K4me3 levels and decreased chromatin accessibility at meiotic genes. Isocitrate dehydrogenase 2 (IDH2), which generates α-KG, and oxoglutarate dehydrogenase (OGDH), which consumes it^35,36^, showed increased and decreased trends, respectively, although with *P* > 0.05 (Supplementary Fig. S15b), indicating a metabolic shift favoring α-KG accumulation. In male PGCs, the expression of major DNA methylation enzymes remained largely unchanged, except for a modest decrease in *Dnmt3a* (*P* = 0.07) and slight upregulation of *Tet1/2/3*, especially *Tet1* with marginal significance (Supplementary Fig. S15c), in line with the decreased DNA methylation level. Similar α-KG elevation and increased *Idh2* expression were observed (Supplementary Fig. S15d, e), coinciding with a global reduction in DNA methylation. Together, these data suggest that IUHG may modulate PGC development through metabolic-epigenetic crosstalk, with distinct regulatory vulnerabilities between sexes.

### The impact of IUHG on meiotic progression in fetal oocytes is sustained

**Fig. 5:**
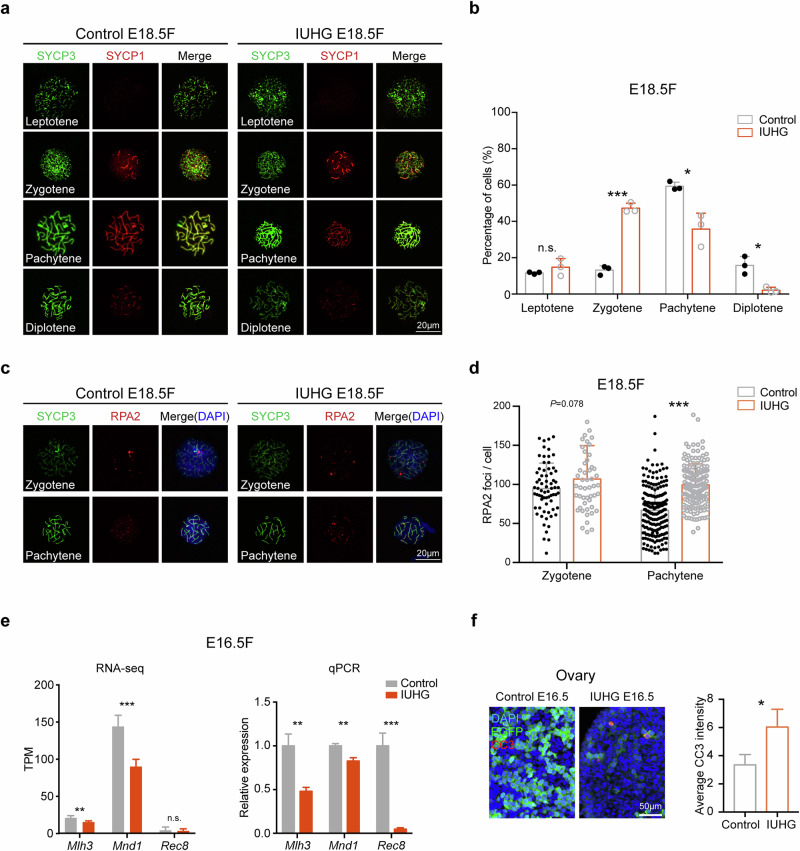
The sustained impact of IUHG on meiotic progression during female PGC development. **a** Immunofluorescence staining of meiotic prophase I markers SYCP1 (red) and SYCP3 (green) in fetal oocytes from control and IUHG mice at E18.5. **b** Quantification of meiotic stages distribution in oocytes from fetuses at E18.5 in the control and IUHG. A minimum of 200 cells was counted per experiment, with three biological replicates conducted. **c** Immunostaining for RPA2 (red) and SYCP3 (green) was performed on control and IUHG oocytes from E18.5 female fetuses. Representative images of oocytes at the zygotene and pachytene stages are shown. **d** Graphs show quantification of RPA2 foci numbers per cell at zygotene and pachytene stages. *n* = 266 biologically independent oocytes for the control group; *n* = 208 biologically independent oocytes for the IUHG group. **e** RNA-seq and qPCR analysis of meiosis-related genes (*Mlh3*, *Mnd1*, and *Rec8*) at E16.5 female PGCs in control and IUHG. Data are summarized from three biologically independent experiments. **f** Cleaved Caspase-3 staining in ovarian sections at E16.5 shows Cleaved Caspase-3 (red), EGFP (green), and counterstained with DAPI (blue) for nuclei under IUHG. Data are summarized from three independent biological replicates, with each replicate collected from a distinct embryo. Data are shown as mean ± SD. Wald’s test calculated by DESeq2 is used in **e** (left); Student’s *t*-test is used in **b**, **d**, **e** (right), **f**; *P < 0.05, **P < 0.01, ***P < 0.001, n.s., not significant.

Transcriptomic and ATAC-seq data indicate that the expression of meiosis-related genes and chromatin accessibility of the promoter region gradually return to near-normal levels in the E16.5 IUHG (Figs. 3g, 4c). To further investigate whether the meiotic process in offspring remains affected under persistent IUHG during late pregnancy, we examined chromosomal behavior in E18.5 oocytes. Spreading results showed that the proportion of oocytes in the zygotene stage was increased, while those in the pachytene and diplotene stages were significantly reduced in E18.5 IUHG fetal oocytes compared to the control groups (Fig. 5a, b).

To assess whether recombination progression was affected, we examined RPA2, a marker for persistent DNA double-strand break (DSB) intermediates^37^. While RPA2 foci levels were comparable between groups at the zygotene stage, a significant increase in RPA2 foci was observed in pachytene-stage oocytes from the IUHG group, suggesting delayed or inefficient DSB repair (Fig. 5c, d). These results indicate that although meiotic initiation occurs, recombination resolution is disrupted under hyperglycemic conditions. In line with this, *Mlh3*, *Mnd1*, and *Rec8*, three essential genes for maintenance of meiotic prophase from the leptotene to diplotene stages^38–40^, are moderately downregulated in the E16.5 fetal oocytes IUHG according to our RNA-seq and qPCR (Fig. 5e). Furthermore, cleaved caspase-3 staining demonstrated the increased apoptosis in the ovarian tissues of the IUHG at E16.5 (Fig. 5f). In summary, these results suggest that IUHG exerts a sustained adverse effect on meiotic progression in fetal oocytes.

### IUHG disrupts the sex differentiation of female and male offspring

**Fig. 6:**
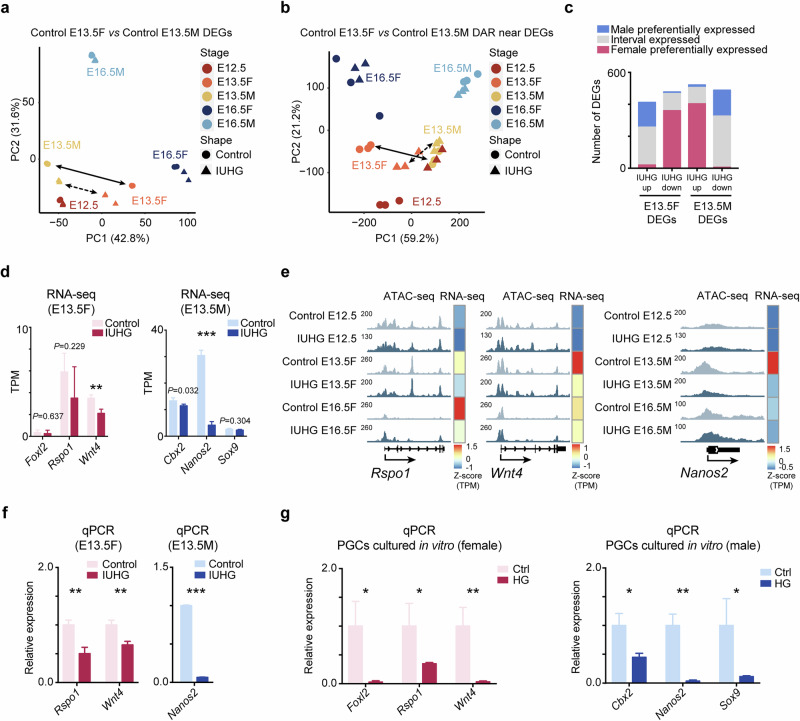
Sex-specific transcriptional and chromatin accessibility alterations in PGCs under hyperglycemic condition. **a**, **b** PCA analysis of DEGs (**a**) and chromatin accessibility (**b**) changes in E13.5 male and female PGCs (E13.5 F vs E13.5 M) under control and IUHG conditions. **c** Barplots showing the numbers of female- and male-preferentially expressed DEGs (upregulated and downregulated) in E13.5 female and male PGCs under control and IUHG conditions. **d** The expression of sex differentiation-related genes at E13.5 PGCs: female regulatory gene network (*Foxl2*, *Rspo1*, and *Wnt4*) and male regulatory gene network (*Cbx2*, *Nanos2*, and *Sox9*) according to RNA-seq analysis (*n* = 3). **e** Genome browser view showing chromatin accessibility at TF binding sites around the *Rspo1*, *Wnt4*, and *Nanos2* loci in fetal PGCs at E12.5–E16.5 development stages in control and IUHG mice. The heatmap shows the expression of those genes. **f** qPCR validation of sex differentiation-related genes (*Rspo1*, *Wnt4*, and *Nanos2*) at E13.5 PGCs shows sex-specific expression differences in control and IUHG (*n* = 3). **g** In vitro cultured female and male PGCs exposed to HG conditions demonstrate altered expression of germline-specific genes (female: *Foxl2*, *Rspo1*, and *Wnt4*; male: *Cbx2*, *Nanos2*, and *Sox9*) compared to controls (*n* = 3). Data are presented as mean ± SD. Wald’s test calculated by DESeq2 is used in **d**; Student’s *t*-test is used in **f** and **g**; *P < 0.05, **P < 0.01, ***P < 0.001.

After sex differentiation, female and male germ cells follow distinct development pathways: by E13.5, female germ cells gradually exit pluripotency and enter meiosis, while male germ cells retain pluripotency and arrest at the G1/G0 phase until birth^26^. Evidence suggests that the sex determination of female germ cells is primarily driven by the induction of meiosis or inhibition of male meiosis-associated pathways^41,42^. Interestingly, PCA and analyses of DEGs and DARs in male and female E13.5 PGCs revealed reduced differences between female and male PGCs under IUHG compared to controls (Fig. 6a, b). GO analysis further highlighted correlations with sex differentiation (Fig. 3f and Supplementary Fig. S6b).

In IUHG E13.5 PGCs, upregulated genes in female PGCs showed male-preferred expression patterns, while in contrast, upregulated genes in male PGCs showed female-preferred expression patterns (Fig. 6c). RNA-seq data show that female-specific genes such as *Foxl2*, *Rspo1*, and *Wnt4* were downregulated in IUHG E13.5 female PGCs, and male-specific genes, including *Cbx2*, *Nanos2*, and *sox9*, were downregulated in IUHG E13.5 male PGCs (Fig. 6d). Incorporating ATAC-seq data, we observed that the chromatin accessibility of *Rspo1* and *Wnt4* decreased in IUHG female PGC group at E13.5, and the chromatin accessibility of *Nanos2* was dramatically reduced in IUHG male PGC group at E13.5 (Fig. 6e), confirming its role in regulating sex-determination genes. The qPCR results confirmed the findings, showing a similar trend of decreased expression levels for the genes analyzed under hyperglycemic conditions (Fig. 6f). These findings were validated in cultured PGCs subjected to high glucose treatments in vitro (Fig. 6g). Notably, *Nanos2* expression decreased in IUHG E13.5 male PGCs, while *Stra8*, a meiotic initiation gene, was significantly upregulated (Fig. 6d–g and Supplementary Figs. S8b, d, S9e). Taken together, these results suggest that IUHG delays sex differentiation by inhibiting key sexual determination factors in female mice or promoting meiosis-related gene expression in male mice.

### DNA methylation, but not open chromatin reprogramming, is severely impaired in male mouse PGCs at E16.5

**Fig. 7:**
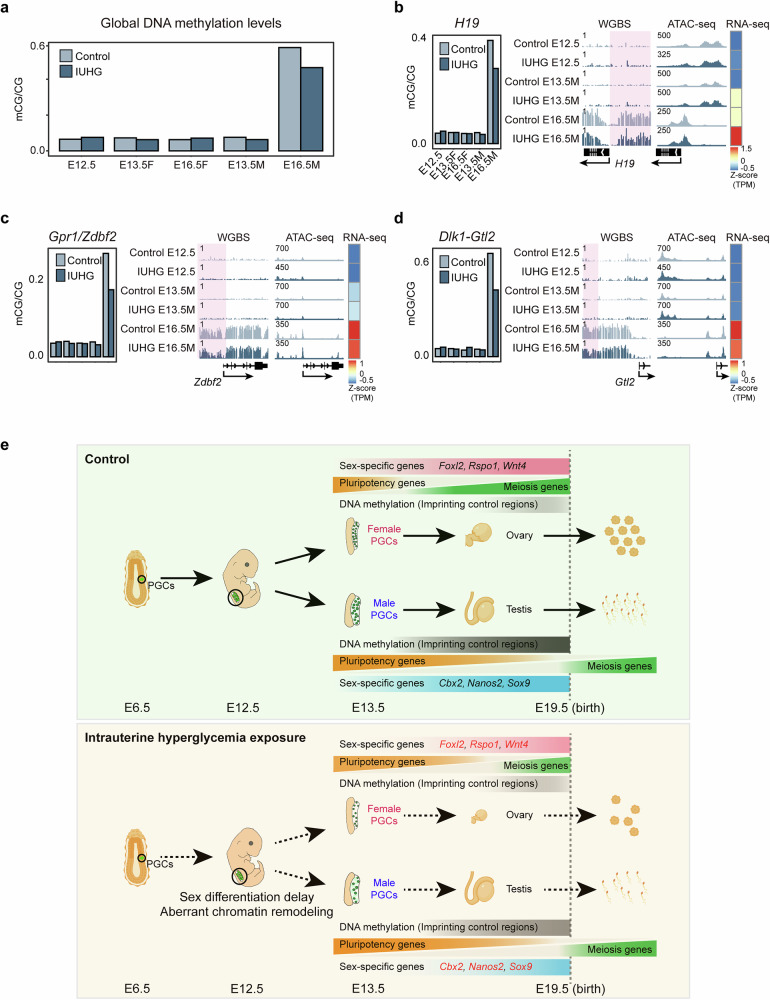
Genome-wide DNA hypomethylation occurred in E16.5 male PGCs exposed to IUHG. **a** Global DNA methylation levels in control and IUHG PGCs across different developmental stages (E12.5, E13.5, and E16.5). Data are summarized from three biologically independent experiments. **b**–**d** DNA methylation, chromatin accessibility (ATAC-seq), and gene expression (RNA-seq) profiles for ICRs. Changes in methylation levels of ICRs (*H19*, *Gpr1/Zdbf2*, and *Dlk1-Gtl2*) across different developmental stages. Genome browser view showing the DNA methylation and ATAC signal enrichment in fetal male PGCs at E12.5, E13.5 and E16.5 in control and IUHG. The heatmap shows the expression of those genes. **e** A schematic summary comparing normal germ cell development (top) to the disrupted development observed under IUHG (bottom). In normal conditions, PGCs undergo proper sex differentiation, with tightly regulated DNA methylation and chromatin remodeling to activate sex-specific and meiosis-related genes. Under hyperglycemic conditions, delayed sex differentiation and aberrant chromatin remodeling result in disrupted expression of pluripotency and sex-specific genes, impairing germ cell maturation.

Epigenetic reprogramming in PGCs is primarily defined by a cycle of global DNA demethylation followed by remethylation^18^. DNA methylation reprogramming is an indispensable process in the development of mammalian PGCs, playing a pivotal role in the erasure and re-establishment of genomic imprinting^43^. To assess the impact of IUHG on methylation patterns in fetal PGCs, we performed WGBS across three independent samples at each time point (Supplementary Fig. S16a). In female PGCs, low methylation levels observed at E13.5 were maintained through E16.5, while male PGCs at E16.5 typically undergo robust de novo methylation (Fig. 7a), consistent with prior studies^18,44^. Global hypomethylation remained comparable in female PGCs during E12.5–E16.5, likely due to postnatal de novo methylation. However, in IUHG E16.5 male PGCs, we observed an obvious global hypomethylation, with methylation levels reduced by ~10% compared to controls (Fig. 7a and Supplementary Fig. S16b, c). Similarly, we observed that the DNA methylation levels of pluripotency-related and meiosis-associated differentially methylated regions (DMRs) were significantly reduced only at IUHG E16.5 male PGCs when compared with the control group, with no notable differences detected at other developmental stages (Supplementary Fig. S16d).

Parental chromosomes are marked by specific epigenetic signatures at imprinting control regions (ICRs), regulated by DNA methylation, to control parent-specific gene expression^45^. Disruptions in these imprinted loci can lead to serious developmental disorders in humans^46,47^. We identified a group of ICRs with altered DNA methylation and mRNA expression patterns in IUHG E16.5 male PGCs, impacting imprinted genes such as *H19*, *Zdbf2*, *Gtl2*, and *Dlk1* (Fig. 7b–d and Supplementary Fig. S16e–g). In IUHG male PGCs, these ICRs exhibited significantly reduced methylation levels, alongside changes in the expression of their associated genes. This indicates that proper ICR methylation is crucial for maintaining imprinted gene expression. It has been reported that DNA methylation plays a crucial role in the epigenetic programming of spermatogonial stem cell differentiation and the regulation of lifelong spermatogenesis^31^. Thus, fetal male PGCs exposed to a high-glucose environment may be affected through alterations in DNA methylation, particularly at ICRs. These changes may be able to disrupt the differentiation of pluripotent cells and the process of meiosis postnatally, thereby impairing spermatogenesis and ultimately influencing the next generation.

## Discussion

Fertility issues are global challenges, driven by multiple factors^48^. The increasing prevalence of IUHG, such as GDM, poses challenges for maternal and child health^49–51^. Increasing evidence highlights the adverse outcomes associated with IUHG, particularly in the reproductive systems of offspring^52^, while the underlying mechanisms await further investigation. In this study, we revealed how IUHG affects fetal germ cell development in sex-specific ways (Fig. 7e). Briefly, offspring embryos directly exposed to IUHG exhibited impaired chromatin remodeling in E12.5 PGCs. In female offspring, IUHG PGCs exhibit developmental delays between E13.5 and E16.5, primarily due to their inability to suppress the pluripotent transcriptional program and initiate meiosis, ultimately leading to a reduced ovarian reserve of oocytes and decreased fertility. This delay is largely attributed to the impaired chromatin accessibility at pluripotency genes as early as E12.5, ahead of gene expression changes. In male offspring, the IUHG group results in significantly reduced sperm concentration. Unlike females, disrupted DNA methylation reprogramming was observed during male PGC development. Both male and female offspring exposed to IUHG exhibited compromised fertility, likely through alterations in DNA methylation and chromatin remodeling in PGCs, respectively. Some of the alterations may even be able to pass to mature gametes and exert intergenerational effects, in line with previous studies that GDM-induced epigenetic alterations can persist into the F2 generation^21,24^. These inherited changes, detectable as early as at E13.5 in F1 PGCs, suggest that IUHG may exert intergenerational effects through disrupted germline epigenetic reprogramming.

IUHG induces distinct epigenetic alterations in female and male PGCs, characterized by chromatin accessibility changes in females and DNA methylation alterations predominating in males. These differences may be attributed to the unique developmental timelines of male and female PGCs^26^. Female PGCs initiate meiosis earlier, a process requiring dynamic chromatin remodeling, which may be more sensitive to metabolic perturbations during this developmental stage^29^. By contrast, male PGCs undergo significant DNA methylation reprogramming during mitotic arrest, a stage that coincides with critical windows of hyperglycemia-induced metabolic stress^18^. Moreover, it is conceivable that sex-specific metabolic states of PGCs during differentiation contribute to these divergent epigenetic outcomes. Hyperglycemia is known to disrupt key metabolic pathways, including glycolysis, the tricarboxylic acid cycle, and the one-carbon metabolism pathway^53^. These disruptions can affect the availability of key metabolites such as S-adenosylmethionine, acetyl-CoA, and α-KG, which serve as cofactors or substrates for DNA and histone-modifying enzymes^54^. During the intrauterine window from E12.5 to E16.5, we found that IUHG exposure elicited similar changes in α-KG levels and the expression patterns of α-KG-related metabolic enzymes in both male and female E13.5 PGCs. Given the multifaceted roles of α-KG in both histone and DNA demethylases, its functional specificity likely depends on the developmental stage and epigenetic reprogramming of the germ cells. Specifically, in female PGCs that initiate meiosis but have not yet begun de novo DNA methylation and thus remain in a hypomethylated state, elevated α-KG level may primarily impairs H3K4me3 deposition. Notably, PRDM9-dependent H3K4me3 is essential for meiotic entry and DSB formation, and its reduction may contribute directly to the observed meiotic defects (Fig. 5a and Supplementary Fig. S14). By contrast, male PGCs at this stage are undergoing extensive remethylation but have not yet initiated meiosis. In this context, increased α-KG may preferentially enhance the activity of TET enzymes, leading to impaired DNA remethylation (Fig. 7a and Supplementary Fig. S15c, d). These sex-specific effects underscore how the interplay between metabolic state and epigenetic remodeling is tightly coordinated with germ cell developmental timing. While our findings reveal a pivotal role for the metabolic-epigenetic axis in mediating the detrimental effects of maternal hyperglycemia on offspring germline development, we cannot exclude the involvement of additional metabolic intermediates and epigenetic regulators in this process. We acknowledge that the precise molecular mechanisms linking metabolic alterations to chromatin-modifying enzyme activity and changes in chromatin accessibility remain incompletely understood. In particular, technical limitations, such as the scarcity of PGCs and current constraints in technical sensitivity, pose significant challenges to directly profiling metabolites in rare cell populations. There is an urgent need for the development of robust ultra-low-input or even single-cell metabolomic approaches to enable precise quantification of global metabolic states in such contexts.

Current clinical management of IUHG primarily targets maternal blood glucose and improves overall glucose metabolism through lifestyle interventions, insulin therapy, or oral hypoglycemic agents^55^. While these strategies can reduce maternal and perinatal complications, their ability to mitigate the long-term reproductive effects of IUHG on offspring remains uncertain^56^. Although direct validation using human fetal germ cells is limited by ethical and logistical challenges, recent clinical studies have reported sex-specific epigenetic and transcriptomic alterations in placental and cord blood samples from GDM pregnancies^57–59^. These changes are enriched in metabolic and hormonal pathways, suggesting early, sex-specific fetal programming. Together, these findings provide translational support for our murine observations and underscore the relevance of epigenetic mechanisms in mediating the long-term effects of IUHG.

Studying the development of PGCs is crucial for better elucidating the molecular mechanisms of diseases. Our findings indicate the potential role of IUHG in disrupting key epigenetic processes in developing germ cells. To date, no specific pharmacological or non-pharmacological interventions directly target the restoration of epigenetic homeostasis or reproductive system development in offspring affected by IUHG. Metabolic pathways linked to epigenetic modifications, such as the one-carbon metabolism and acetyl-CoA pathways, could serve as potential therapeutic targets^60^. Future intervention strategies could involve dietary supplementation (e.g., folate, vitamin B12, or acetyl donors), pharmacological modulators of epigenetic enzymes, or small molecules designed to correct specific chromatin remodeling defects^61,62^. While the study identifies epigenetic changes in germ cells, the precise mechanisms by which hyperglycemia drives these alterations remain unclear. This study highlights the need for a paradigm shift in the management of IUHG, moving beyond maternal glucose control to include targeted interventions for offspring health. Interventions targeting PGCs to achieve clinical therapeutic goals remain a subject for further research. In conclusion, our study provides valuable insights into the developmental, transcriptional, and epigenetic changes in fetal PGCs exposed to IUHG.

## Materials and methods

### Establishment of a mouse model

All animal experiments were approved by the Animal Care and Use Committee of Fudan University. Mice were maintained on a 12-h light and 12-h dark cycle in a constant temperature (20–25 °C) and humidity levels (30%–70%). To specifically model gestational hyperglycemia while minimizing confounding factors such as maternal obesity or pre-gestational diabetes, we used STZ (Sigma) to induce diabetes on day 3.5 of pregnancy. STZ rapidly and selectively ablates pancreatic β cells due to its short half-life, allowing precise control of hyperglycemia onset. Importantly, STZ-injected dams without elevated blood glucose showed no fetal germ cell phenotype, confirming that the observed effects were attributable to hyperglycemia rather than STZ itself. The detailed procedures are described below. Female mice aged eight weeks were mated with wild-type or *Oct4***-***EGFP* male mice. The presence of a vaginal plug the following morning was defined as E0.5 of pregnancy. Gestational mice were randomly divided into the control group and the IUHG. After a 12-h fast, mice in the IUHG were intraperitoneally injected with STZ at 120 mg/kg body weight on day 3.5 of pregnancy. At E6.5 of pregnancy, the blood glucose concentration was measured via the tail vein using a glucometer (Roche Diagnostics Accu-Chek, Germany). A blood glucose level of more than 14 mM was used as the inclusion criterion for diabetes. Blood glucose levels were monitored throughout the prenatal period to confirm the occurrence and progression of diabetes.

### Fetus collection and PGC isolation

After mating, it was defined as E0.5 according to the day of the presence of a vaginal plug. On E12.5, E13.5, E16.5, E17.5, and E18.5, pregnant mice were humanely euthanized by cervical dislocation, and embryos were collected for statistical phenotyping and subsequent experiments. After birth, the ovaries of female mice at different ages (P1, P8, 12 W, 32 W) and testes of male mice (8-week-old) were collected for further analysis.

Embryonic gonads were collected at E12.5, E13.5, and E16.5, with female and male gonads distinguished either by Sry-primer PCR identification or distinct morphological features. PCR was performed using the following primers: forward: ATTTATGGTGTGGTCCCGTGGTGAG; reverse: GTATGTGATGGCATGTGGGTTCCTG. Gonads were enzymatically digested into single-cell suspensions using 0.25% trypsin-EDTA at 37 °C for 5 min. The resulting cells were sorted on a BD FACS Aria II flow cytometer based on EGFP positivity. PGCs were sorted directly into PBS for ATAC-seq or lysed and immediately frozen in liquid nitrogen for subsequent RNA-seq and WGBS.

### IHC

Fresh mouse ovary tissues were fixed in 4% paraformaldehyde and embedded in an optimal cutting temperature compound (Sakura, 4583). Serial sections near the largest section (ovary center) were sliced by a microtome at 5 μm and counted (Leica, CM 1950). The samples were subjected to routine procedures. The sections were incubated with Anti-MVH (Abcam, ab284611).

### Hematoxylin and eosin (H&E) staining

Fresh samples from the mouse ovary and testis were fixed in 4% paraformaldehyde overnight at 4 °C. Samples were dehydrated and embedded in paraffin. A microtome sliced the samples by a microtome at 5 μm thick (Leica, CM 1950). The sections were routinely deparaffinized and stained with H&E following standard protocols.

### Quantification of ovarian follicles

Quantification of ovarian follicles was performed following established protocols^63^. Paraffin-embedded ovaries were serially sectioned at a thickness of 5 μm and analyzed using either IHC or H&E staining for morphological assessment. For IHC analysis, serial sections near the largest ovarian cross-section (center) were prepared, with the fourth section being examined to count ovarian follicles. For H&E staining, follicles at different developmental stages, including primordial, primary (type 3 and type 4), pre-antral (type 5), and antral follicles (type 6 and type 7), were identified and quantified in every fifth section throughout one ovary. This classification was based on well-established criteria described by Pederson and Peters^64^. Only follicles with a visible oocyte nucleus were counted in each section. To estimate the total number of follicles in an ovary, cumulative counts were multiplied by a correction factor of 5.

### Chromosome spreads

The experimental protocol for chromosome spreading was conducted as previously described^65^. Dissected ovarian tissues from E13.5 and E18.5 mouse embryos were placed in a hypotonic solution (17 mM trisodium citrate dihydrate, 50 mM sucrose, 5 mM EDTA, 0.5 mM DTT, 30 mM Tris-HCl, 0.5 mM protease inhibitor PMSF, pH 8.2) and cultured for 15 min at 37 °C. Using forceps under a stereomicroscope, the ovaries were gently dispersed in a 100 mM sucrose solution (pH 8.2). Finally, the cell suspension was applied to adhesive slides and fixed in 1% paraformaldehyde (containing 0.2% Triton X-100) solution in a humidified chamber for 2 h. After drying, the slides can be stored at –80 °C for short-term preservation and used for subsequent experiments. The slides were immersed in 0.5% Triton X-100 for 15 min, then blocked with 5% BSA solution at room temperature for 1 h and stained with primary antibodies (Anti-SYCP1: Abcam, ab303520; Alexa Fluor®488 Anti-SYCP3: Santa, sc-74569; RPA2: Abcam, ab76420) at 4 °C overnight. Slides were washed three times with PBS for 5 min each, followed by incubation with the secondary antibody (Anti-Cy3: Abcam, ab6939) for 1 h at room temperature. Next, the slides were washed three times with PBS for 5 min each, followed by drying and mounting. Finally, the slides were imaged using a laser confocal microscope (Nikon ECLIPSE Ti2, Japan), and the data were subjected to statistical analysis by Nikon NIS software.

### Immunofluorescence staining of embryonic gonads

Mouse gonads were dissected at E12.5, E13.5, and E16.5, followed by fixation in 4% paraformaldehyde at 4 °C. After fixation, tissues were dehydrated in 30% sucrose, embedded in OCT compound (Sakura), and sectioned at 10-μm thickness using a cryomicrotome (Leica, CM 1950). For immunofluorescence staining, sections were incubated with primary antibodies against (Ki67: Abcam, ab16667; Cleaved caspase-3: Cell Signaling Technology, 9661S; SALL4: PPMX Perseus Proteomics, PP-PPZ0601-00; STRA8: Abcam, ab49602; EGFP: Abcam, ab13970; H3K4me3: Cell Signaling Technology, 9751 T; H3K27ac: Abcam, ab4729), followed by appropriate fluorophore-conjugated secondary antibodies (Anti-Cy3: Abcam, ab6939 and ab97035; Anti-488: Abcam, ab150173). Images were captured using a confocal laser scanning microscope (Nikon ECLIPSE Ti2, Japan).

### Bulk RNA-seq and data analysis

Total RNA was extracted from female gonads at E13.5 (4 pairs of female gonads per sample, 3 samples for each group) with the Universal RNA Extraction CZ Kit (RNC643, ONREW) according to the manufacturer’s instructions. Samples used for Bulk RNA-seq library construction were listed in Supplementary Table S4. RNA quality was analyzed using Qubit 4.0 (Invitrogen), and quality was examined by electrophoresis on a denaturing agarose gel. RNA libraries were prepared using the VVAHTS® Universal V8 RNA-seq Library Prep Kit for Illumina (NR605-0, Vazyme), followed by sequencing using the Illumina NovaSeq 6000 platform with the 150 paired-end sequencing strategy. Enrichment of mRNA, library construction, sequencing and data analysis were performed by Shanghai Xu Ran Biotechnology Co., Ltd.

Raw bulk RNA-seq data were quality-checked with FastQC (v0.12.1) and trimmed for adapters and low-quality bases using Trim Galore (v0.6.10). Clean reads were aligned to the mouse genome (mm9) using HISAT2 (v2.2.1), and sorted BAM files were generated via SAMtools (v1.6). StringTie (v2.1.7) was used for transcript assembly and quantification, guided by GTF annotation, to obtain gene-level raw counts and FPKMs. Differential expression analysis was performed using the DESeq2 R package (v1.42.1). Genes with fewer than 10 total counts were excluded, and normalization was applied using the median-of-ratios method. A design formula including the experimental condition (IUHG vs Control) was specified. DESeq2 estimated size factors and dispersions, and conducted Wald tests based on the negative binomial distribution. Genes with FDR-adjusted *P* < 0.05 and |log₂ fold change| ≥ 1 were considered differentially expressed. DEGs were visualized using ggplot2 and pheatmap, and used for subsequent pathway analysis.

Gene Set Variation Analysis (GSVA) analysis was performed on the FPKM expression matrix derived from bulk RNA-seq data^66^. The top 10,000 most variable genes across samples were retained for the analysis. Gene sets corresponding to KEGG and GO Biological Process pathways were downloaded from the Molecular Signatures Database (MSigDB, collections: m5.go.v2024.1.Mm.symbols.gmt)^67^. The GSVA scores were computed using the “gsva()” function in the GSVA R package (version 1.50.5), with default parameters. Differential pathway activity between experimental groups was evaluated using the limma package.

### RNA-seq (Smart-seq2) and data analysis

Total RNA was extracted from fetal PGCs (collected from E12.5, E13.5, and E16.5; 20–30 fetuses per sample, 3 samples for each group) with the Universal RNA Extraction CZ Kit (RNC643, ONREW) according to the manufacturer’s instructions. Samples used for RNA-seq library construction were listed in Supplementary Table S4. RNA quantity was analyzed using Qubit 4.0 (Invitrogen) and quality was examined by electrophoresis on a denaturing agarose gel. Total RNA was used as input material. cDNA synthesis and amplification were performed by Single Cell Full Length mRNA-Amplification Kit (N712-03, Vazyme), and purification by VAHTS DNA Clean Beads (N411-02, Vazyme). Sequencing libraries were prepared by following the TruePrep® DNA Library Prep Kit V2 (TD502-02, Vazyme) user manual. Sequencing was carried out using the Illumina Novaseq 6000 platform with the 150 paired-end sequencing strategy. Enrichment of mRNA library construction and sequencing were performed by Shanghai Xu Ran Biotechnology Co., Ltd.

RNA-seq data quality assessment was conducted using FastQC. Adapters and low-quality sequencing reads were removed using Trim Galore. The filtered sequencing reads were aligned to the mouse reference genome (Mm39) using the STAR two-pass mapping mode^68^. Only uniquely mapped reads were retained for subsequent analyses. For differential gene expression analysis, the featureCounts tool^69^ generated a gene expression quantification matrix, followed by differential expression analysis using the R package DESeq2^70^, with thresholds set at absolute fold change > 2 and *P* < 0.01. GO enrichment analysis was performed using clusterProfiler^71^ and mouse annotation org.Dr.Mm.db, retaining biological processes with *P* < 0.05 for presentation.

### RNA isolation and qPCR

Total RNA extracted and cDNA synthesized protocols were referred to the RNA sequencing section. qPCR was performed using QuantStudio 5 (Life Technologie) with commercial primers generated for the system. The expression of target genes was normalized to that of the reference gene (*Gapdh*). The primers used in this study are listed in Supplementary Table S5.

### In vitro expansion of cultured E12.5 PGCs with high glucose

PGC culture was performed following the method described in the previous report^72^. In brief, E12.5 gonads (male and female) carrying the *Oct4-EGFP* transgene were dissected and digested into a single-cell suspension. The cell mixture was cultured in GMEM (containing 10% KSR, 0.1 mM NEAA, 1 mM sodium pyruvate, 0.1 mM 2-mercaptoethanol, 100 U/mL penicillin, 0.1 mg/mL streptomycin, 2 mM L-glutamine, 2.5% FBS, 100 ng/mL SCF, 10 μM Forskolin, and 10 μM Rolipram) as the control group. In the HG-treated group, 25 mM glucose anhydrous was added to the medium^22^. After 24 h of in vitro culture, PGCs were sorted and analyzed by FACS.

### ATAC-seq and data analysis

The EGFP-positive PGCs obtained through FACS were washed with PBS. ATAC-seq was performed using the Hyperactive ATAC-Seq Library Prep Kit. Briefly, 5000–10,000 cells were collected and lysed using lysis buffer. The DNA was extracted and amplified for 15 cycles to produce libraries for sequencing. The ATAC-seq libraries were sequenced on the Illumina platform (Illumina, USA). Three biological replicates were analyzed. Samples used for ATAC-seq library construction were listed in Supplementary Table S6.

Adapters and low-quality sequencing fragments from the raw data were removed using Trim Galore. The processed sequencing reads were aligned to the mouse reference genome using Bowtie2. Duplicate sequencing reads were eliminated using Picard MarkDuplicates (http://broadinstitute.github.io/picard/), and reads mapped to the mitochondria were also removed. Due to the Tn5 transposase cutting characteristics, filtered reads were shifted + 4 bp for the positive strand and − 5 bp for the negative strand. Subsequently, ATAC-seq peaks were identified using MACS2^73^ with the following parameters: -q 0.01 --shift -75 --extsize 150 --nomodel --keep-dup all --call-summits. The normalized ATAC-seq signal was generated using bamCoverage with parameters: -binSize 10 --normalizeUsing RPKM -extendReads. DARs at various developmental stages were identified using DiffBind^74^, retaining those with FDR < 0.001 for further analysis.

### Identification of key TFs

Key TFs were predicted using ANANSE^75^ and AME^76^ tools. Genes associated with pluripotency and meiosis were downloaded from the GO database and defined as genes of interest. One criterion was the number of these target genes regulated by TFs predicted by ANANSE. Another criterion involved TF binding motifs enriched in epigenetically dynamic regions near the genes of interest, identified by AME. TFs that ranked highly by both criteria were defined as key TFs.

### α-KG measurement

Samples were extracted from the gonads at E13.5. The α-KG was measured by an α-KG assay kit (MET-5131, Cell Biolabs) following the manufacturer’s instructions. Colorimetric signal was read using the Elx808 microplate reader instrument; the obtained values (two technical replicates per biological replicate) were normalized on total protein extract; *n* = 4 biological replicates per group, each comprising 20–30 pairs of gonads.

### WGBS and data analysis

The EGFP-positive PGCs obtained through FACS were washed with PBS. DNA was isolated from the cells by using QIAamp® DNA Micro Kit (QIAGEN). Genomic DNA was subjected to bisulfite conversion using EpiArt® DNA Methylation Bisulfite Kit (Vazyme) following the manufacturer’s instructions. Libraries for sequencing were prepared using 100–200 ng of genomic DNA by EpiArt® DNA Methylation Library Kit. Libraries were purified for DNA fragments between 100–200 bp. Library quality was assessed on the Agilent 5400 system (Agilent, USA) and then amplified by the PCR system. Pair-end sequencing of samples was performed on the Illumina platform (Illumina, USA). Three biological replicates were analyzed. Samples used for WGBS library construction were listed in Supplementary Table S7.

Raw sequencing reads from WGBS were processed using the integrated methylpy software package^77^. First, methylpy indexed the bisulfite-converted genome sequence, and alignment was conducted in paired-end mode using Bowtie2 as the alignment tool. Then, the call-methylation-state function within methylpy was used to calculate the methylation state of all cytosines across the genome. Finally, biological replicates from different samples were merged for further analysis.

To identify DMRs, we used the DMRfind function in methylpy to detect CG DMRs across all samples. Briefly, DMRfind employs a permutation-based root mean square test of goodness of fit to identify DMSs. DMSs within 250 bp were merged into DMRs. DMRs with FDR < 0.01 and at least five DMSs were retained for downstream analysis. ChIPseeker^78^ was then used to annotate DMRs by aligning them with mouse genome annotations to identify their presence in functional regions such as promoters and enhancers.

To investigate the impact of DNA methylation on gene expression, StringTie^79^ was used for gene expression quantification (FPKM). Genes were divided into three groups based on expression levels, with each group containing 3000 genes of high, medium, and low expression. DNA methylation levels in regulatory regions (5 kb upstream of transcription start sites, gene bodies, and 5 kb downstream of transcription end sites) for each group were visualized using the ComputeMatrix and plotProfile functions in DeepTools^80^.

### Statistical analysis

All analyses were performed using SPSS software version 26 (Chicago, IL, USA) and GraphPad Prism version 7. Statistical comparisons were performed using Student’s *t*-test. Data are presented as the mean ± SD. All experiments were repeated at least three times independently. *P* < 0.05 was considered changes statistically significant (*P < 0.05, **P < 0.01, ***P < 0.001, n.s., not significant). Bioinformatics analysis for RNA-seq, ATAC-seq, and WGBS data was performed according to ENCODE standard pipelines.

## Supplementary information

Supplemental Figures

Supplemental Tables

## Data Availability

The data that support the findings of this study are available from the corresponding author upon reasonable request. The generated and analyzed datasets in the current study are available in the GEO with the accession numbers GSE287234 (RNA-seq), GSE286574 (ATAC-seq), and GSE287312 (WGBS).
