# Supplementary material for: Intrauterine hyperglycemia impairs mouse primordial germ cell development and fertility by sex-specific epigenetic reprogramming interference
Source: Cell Discov. 2025 Sep 9;11:74. doi: 10.1038/s41421-025-00821-0 (PMC12417548; doi:10.1038/s41421-025-00821-0)
Supplement: Supplementary file 1 — Supplemental Figures [file 41421_2025_821_MOESM1_ESM.pdf]

Intrauterine hyperglycemia impairs mouse primordial germ cell development and fertility by sex-specific epigenetic reprogramming interference

Jiangshan Cong[1,7,8], Qing Li[2,3,8,*], Yangyang Li[4,8], Minghao Li[1,7,8], Yan Shi[1,7], Peiran Hu[1,7], Xidi Yin[3], Qianyun Zhang[3], Jianzhong Sheng[4,5], Jinsong Li[2,3], Guolian Ding[1,7,*], Yu Zhang[1,7,*], Hefeng Huang[1,4,5,6,7,9,*]

These authors contributed equally: Jiangshan Cong, Qing Li, Yangyang Li, Minghao Li

Correspondence: liqing2015@sibcb.ac.cn (Q.L.), dingguolian@fudan.edu.cn (G.D.), zhang_yu_sfy@fudan.edu.cn (Y.Z.), huanghefg@hotmail.com (H.H.).

The PDF file includes:

Supplementary Figs. S1 to S16

Captions Supplementary Tables S1 to S7

Supplemental Figures

Supplementary information, Fig. S1

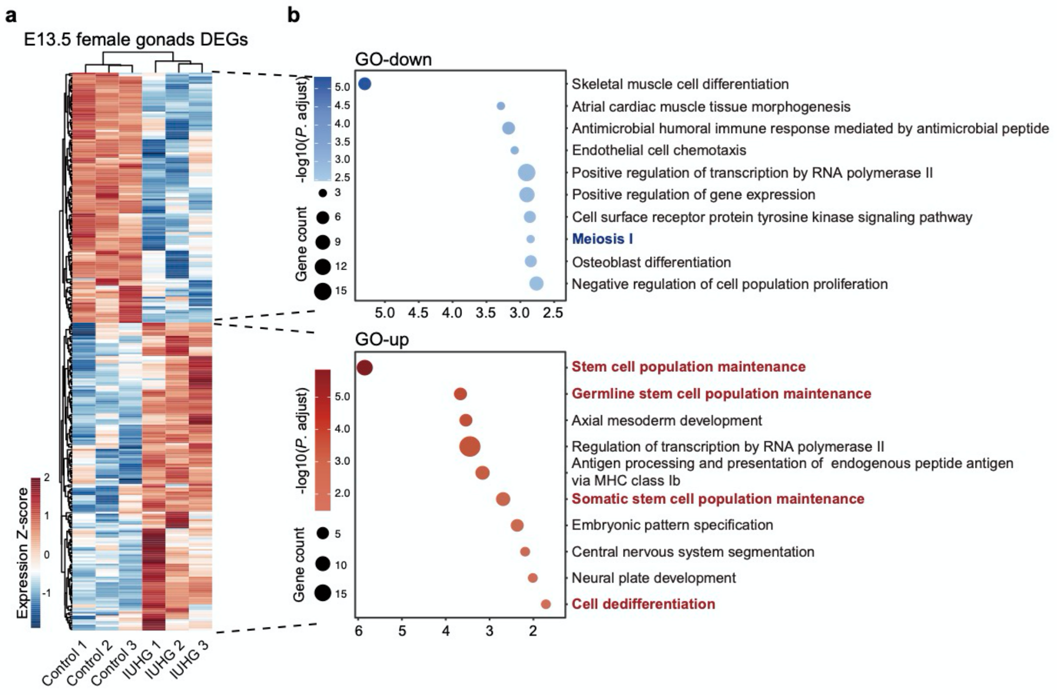

Supplementary information, Fig. S1: GO enrichment analysis of DEGs in E13.5 female gonads.

(**a**) Heatmap shows the normalized expression levels of DEGs in E13.5 female gonads from IUHG and control group by bulk RNA-seq (*n* = 4 pairs of female gonads per sample, *n* = 3 samples per group). (**b**) Bubble chart showing the GO enrichment results for DEGs in E13.5 female gonads.

Supplementary information, Fig. S2

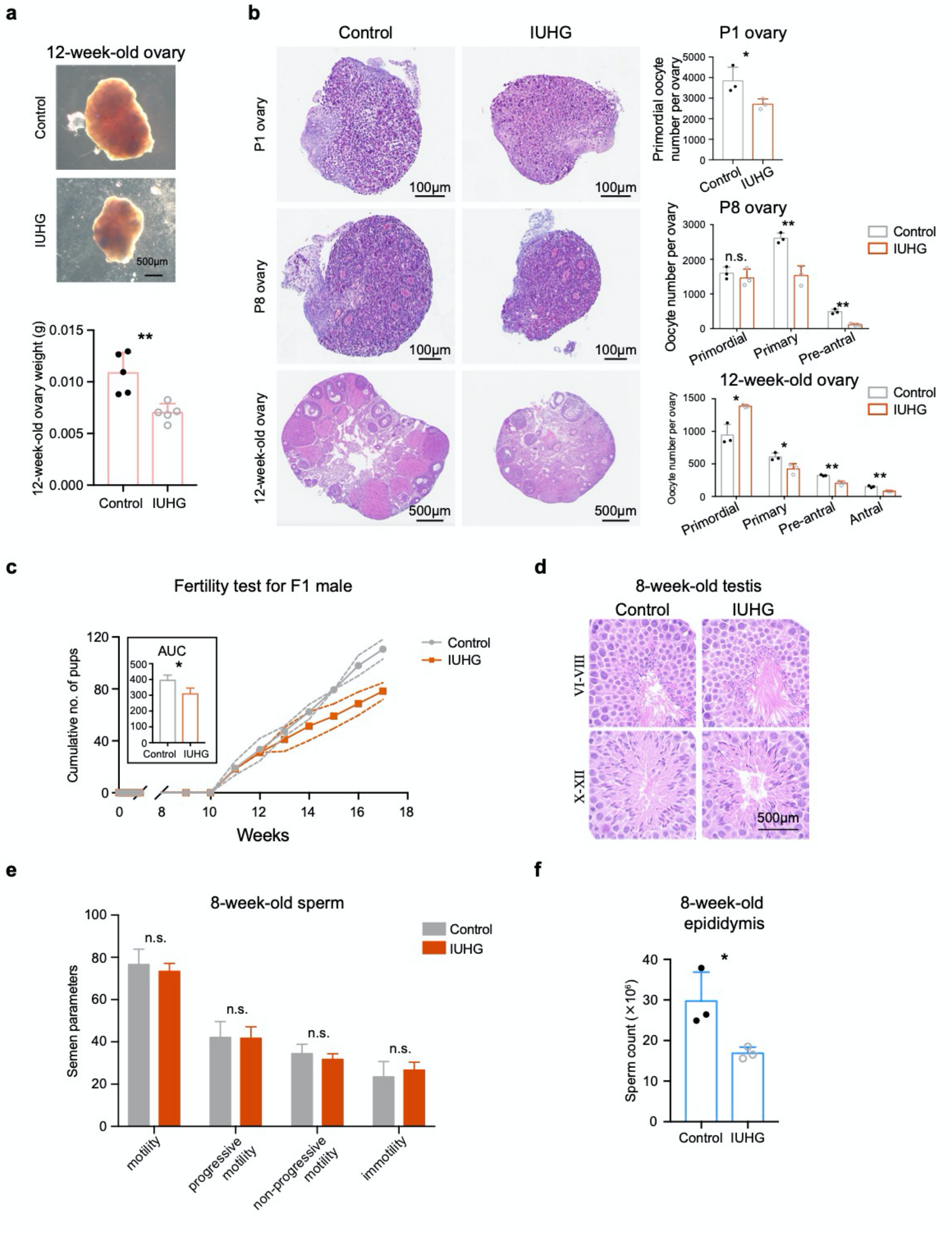

Supplementary information, Fig. S2: Maternal hyperglycemia impairs germ cell development.

(**a**) Stereomicroscope image showing the size and weight of the ovaries in 12-week-old mice from control and IUHG female offspring groups (*n* = 5 per group). (**b**) Left, HE staining was performed to examine the ovarian tissue morphology of control and IUHG

mice at P1, P8, and 12 weeks of age. Right, statistical analysis was performed to detect the number of oocytes at various developmental stages in the ovary. Each group included three biological replicates. (**c**) Fertility test of male offspring, $n = 3$ per group. (**d**) Morphological analysis of testicular sections from control and IUHG male offspring groups was performed at 8 weeks of age. (**e**) Sperm motility in epididymal tissue from control and IUHG male offspring groups at sexual maturity using CASA (Computer-Assisted Sperm Analysis). Each group included three biological replicates. (**f**) Sperm concentration in the epididymis of 8-week-old mice was measured in both the control and IUHG groups. Each group included three biological replicates. All error bars as mean ± SD. Significance was calculated using an unpaired two-sided Student's *t*-test (**a**, **b**, **c**, **f**); $^{*}P < 0.05$, $^{**}P < 0.01$, n.s.: not significant.

Supplementary information, Fig. S3

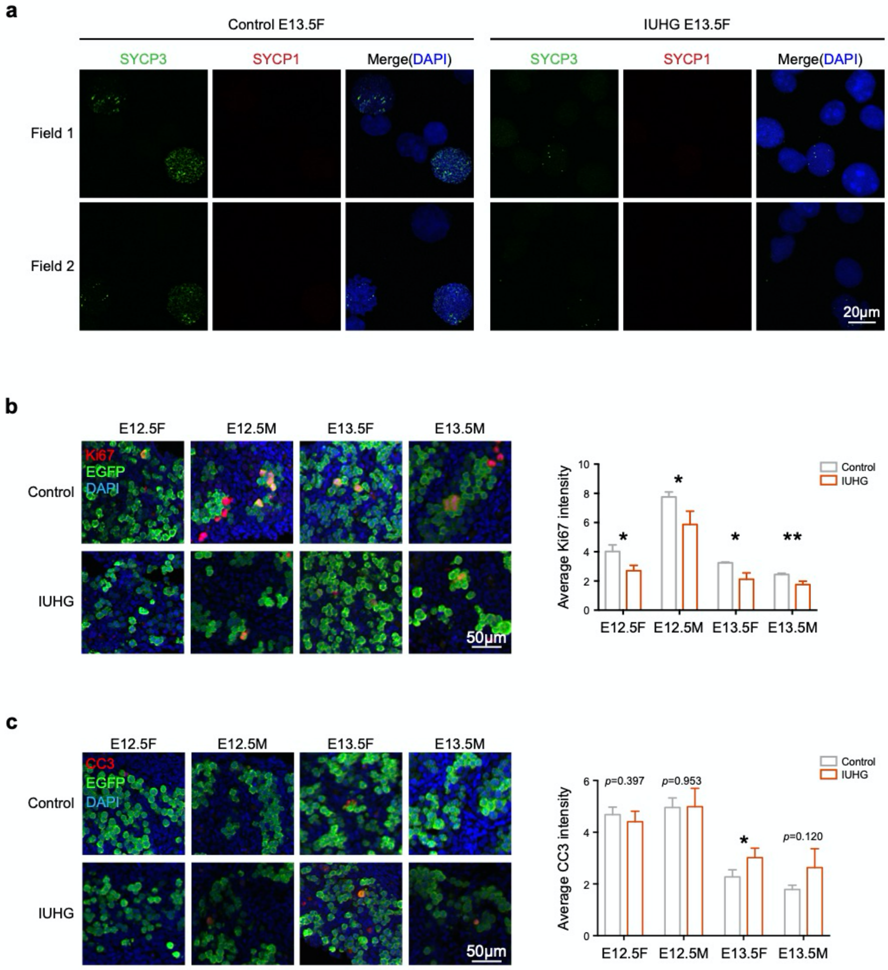

Supplementary information, Fig. S3: Meiotic entry status, proliferation, and apoptosis of E13.5 female germ cells under intrauterine hyperglycemia conditions. (**a**) Immunofluorescence staining of SYCP3 (green), SYCP1 (red), and DAPI (blue) in germ cells from E13.5 female gonads under control and IUHG conditions. Representative images from two different microscopic fields (Field 1 and Field 2) are shown for each condition to illustrate the expression patterns. Scale bar: 20 μm. (**b**) Ki67 signals were significantly reduced under hyperglycemia at both E12.5 and E13.5 stages in male and female gonads (n = 3 slides, n = 3 mice per group). Scale bar: 50 μm. Quantification confirms statistically significant reductions. (**c**) Cleaved Caspase-3 (CC3) staining showed a significant increase only in E13.5 female gonads, indicating sex-specific apoptosis induction under intrauterine hyperglycemia (n = 3 slides, n = 3 mice per group). Scale bar: 50 μm. All error bars as mean ± SD. Significance was calculated using an unpaired two-sided Student's t-test (**b**, **c**); $*P < 0.05$, $**P < 0.01$.

Supplementary information, Fig. S4

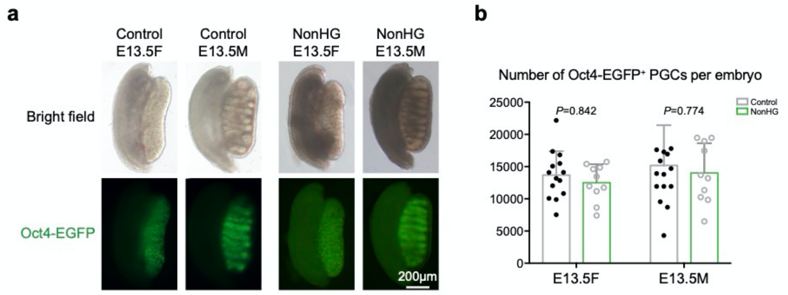

Supplementary information, Fig. S4: Phenotypic analysis of E13.5 gonadal ridges in STZ-injected mice without hyperglycemia.

(**a**) Bright-field and Oct4-EGFP fluorescence images of gonads at E13.5 in control and NonHG (not IUHG) by fluorescence microscopy. Scale bars: 200 µm. (**b**) Average number of Oct4-EGFP^{+} PGCs in whole gonads at E13.5, both in control and NonHG group (*n* = 10 - 16 pairs of gonads per group). Error bars as mean ± SD. Significance was calculated using an unpaired two-sided Student's *t*-test.

Supplementary information, Fig. S5

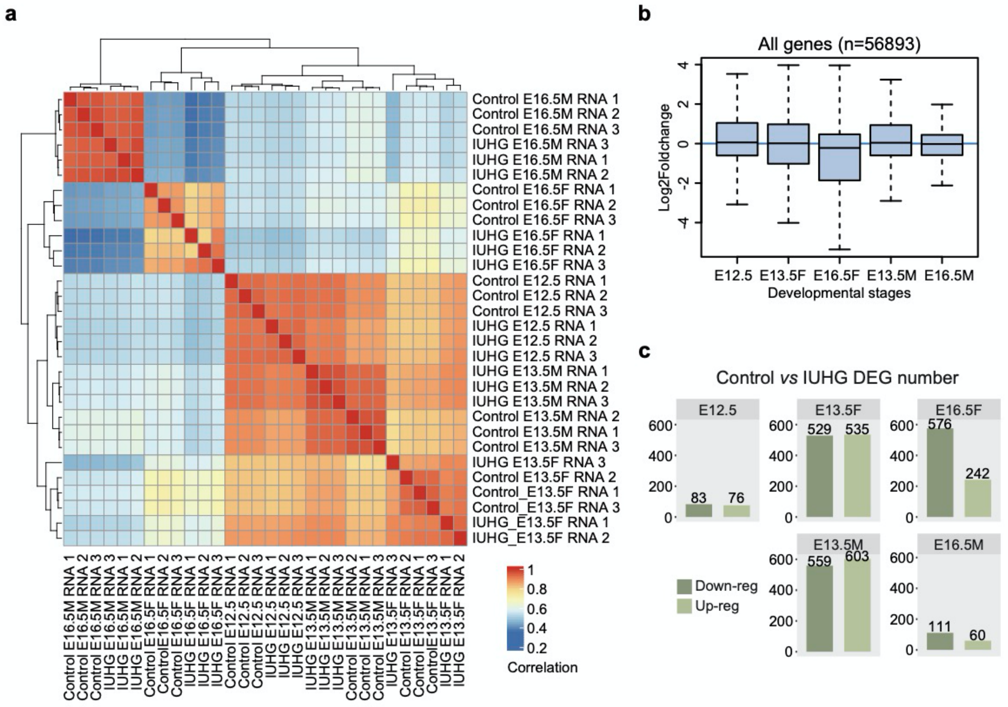

Supplementary information, Fig. S5: Transcriptomic analysis of DEGs in PGCs between control and IUHG offspring.

(**a**) Heatmap showing sample clustering based on transcriptomic similarity across different developmental stages between control and IUHG groups. The clustering demonstrates a clear separation among developmental stages, with IUHG conditions exhibiting distinct transcriptomic profiles compared to controls. (**b**) Box plot illustrating the overall gene expression levels (n = 56,893) across different developmental stages between control and IUHG groups. (**c**) Barplot showing the number of DEGs observed in IUHG groups compared to controls across different developmental stages, categorized into upregulated and downregulated genes.

Supplementary information, Fig. S6

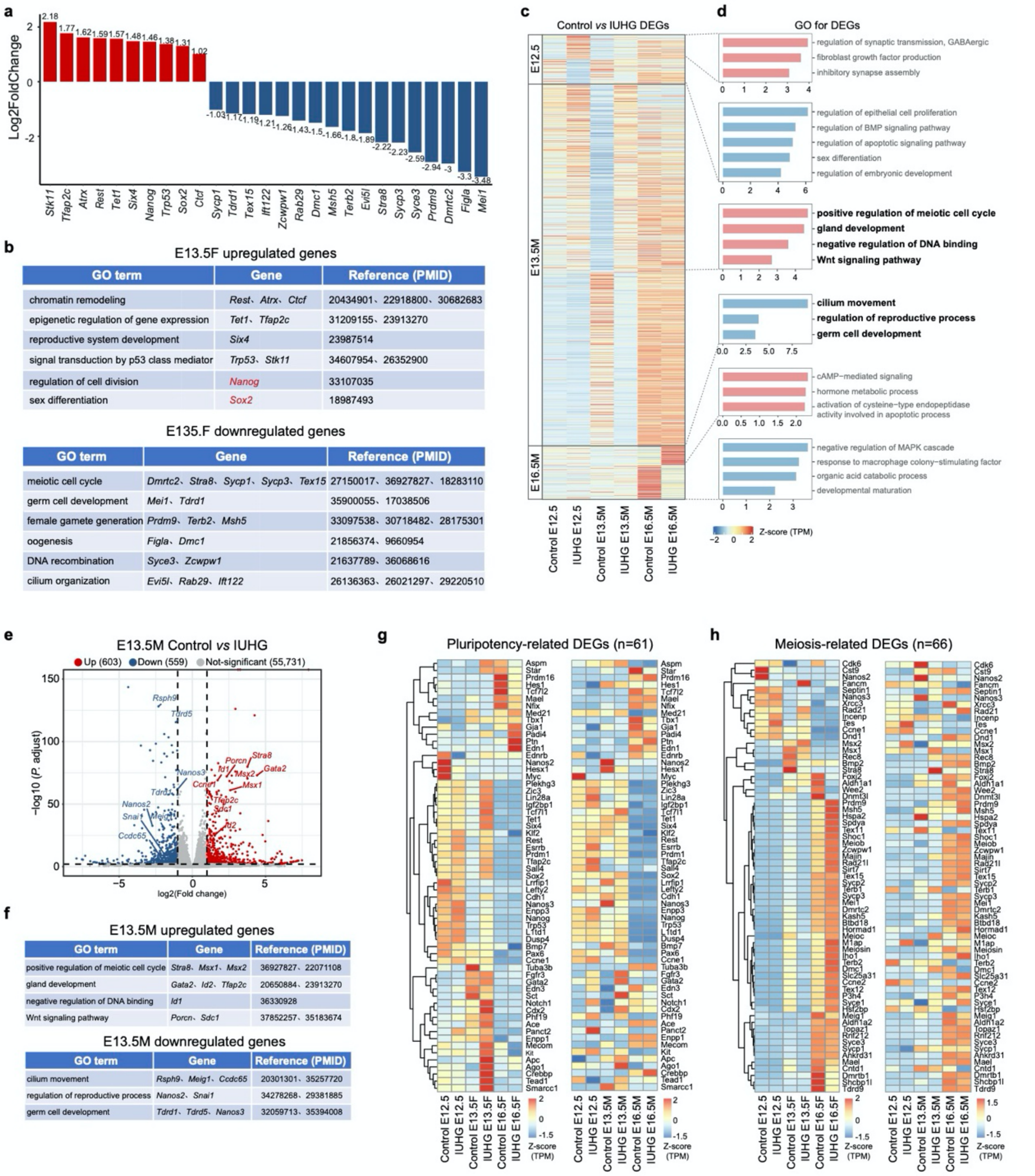

Supplementary information, Fig. S6: Transcriptomic alterations in female PGCs exposed to intrauterine hyperglycemia at E13.5.

(**a**) Expression fold-change of selected DEGs in IUHG E13.5 female PGCs compared to the control group. (**b**) GO enrichment analysis of upregulated and downregulated DEGs highlights their respective functions and representative genes, along with supporting references in IUHG E13.5 female PGCs. (**c**, **d**) Gene expression profiles (**c**) and GO terms (**d**) for upregulated (red) and downregulated (blue) genes in control and IUHG groups at E12.5, E13.5 male (E13.5M), and E16.5M PGCs by RNA-seq. (**e**)

Volcano plot depicting DEGs in IUHG male PGCs at E13.5 compared to the control group. Upregulated genes are shown in red, downregulated genes in blue, and genes with no significant change in grey. (**f**) GO enrichment analysis of upregulated and downregulated DEGs highlights their respective functions and representative genes, along with supporting references in IUHG E13.5 male PGCs. (**g**, **h**) Heatmap showing the expression levels of DEGs related to pluripotency (**g**) and meiosis (**h**) across different developmental stages under control and IUHG conditions.

Supplementary information, Fig. S7

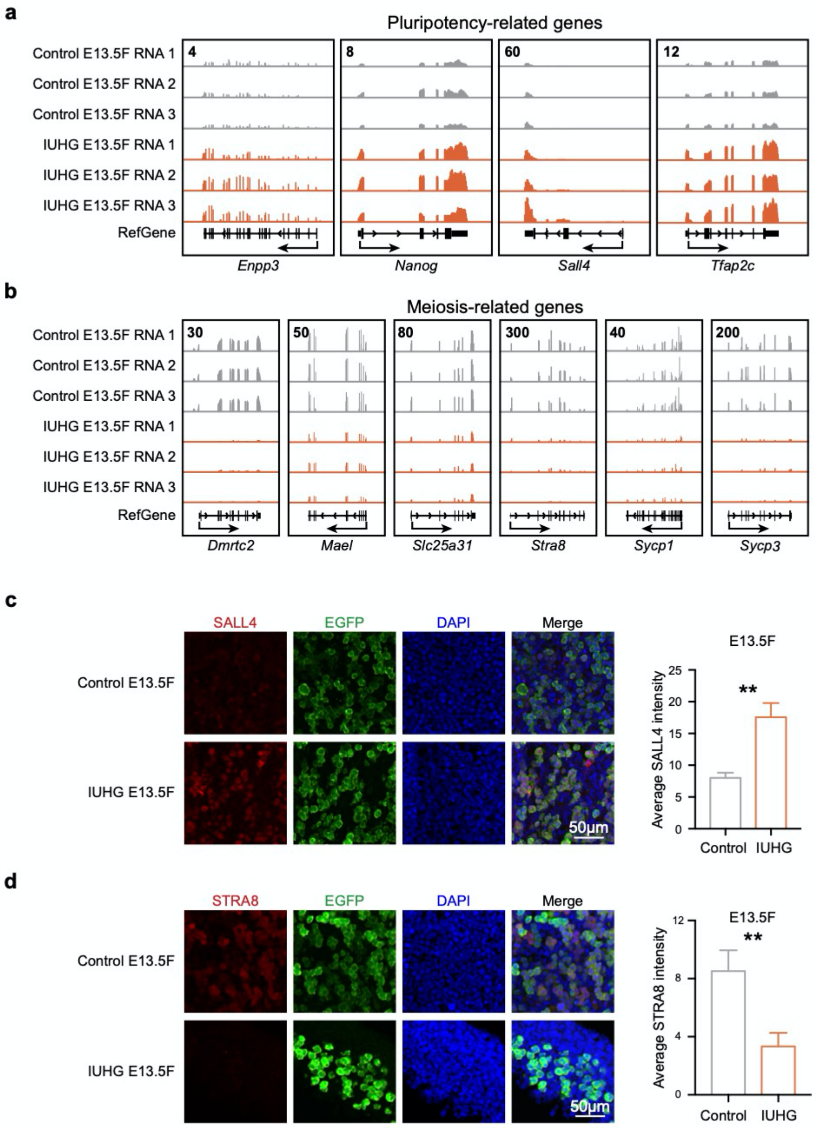

Supplementary information, Fig. S7: Transcriptomic and immunofluorescence evidence for dysregulated pluripotency and meiosis in E13.5 female gonads under intrauterine hyperglycemia.

(**a**, **b**) Genome browser view showing the expression of pluripotency-related genes (*Enpp3*, *Nanog*, *Sall4*, and *Tfap2c*) (**a**) and meiosis-related genes (*Dmrtc2*, *Mael*, *Slc25a31*, *Stra8*, *Sycp1*, and *Sycp3*) (**b**) in control and IUHG group female PGCs at E13.5. Three biological replicates per group. (**c**, **d**) Immunofluorescence staining and quantitative analysis of SALL4 (pluripotency-related, **c**) and STRA8 (meiosis-related, **d**) expression in E13.5 female mouse gonads (n = 3 slides, n = 3 mice per group). All

error bars as mean ± SD. Significance was calculated using an unpaired two-sided Student's *t*-test (**c**, **d**); $**P < 0.01$.

Supplementary information, Fig. S8

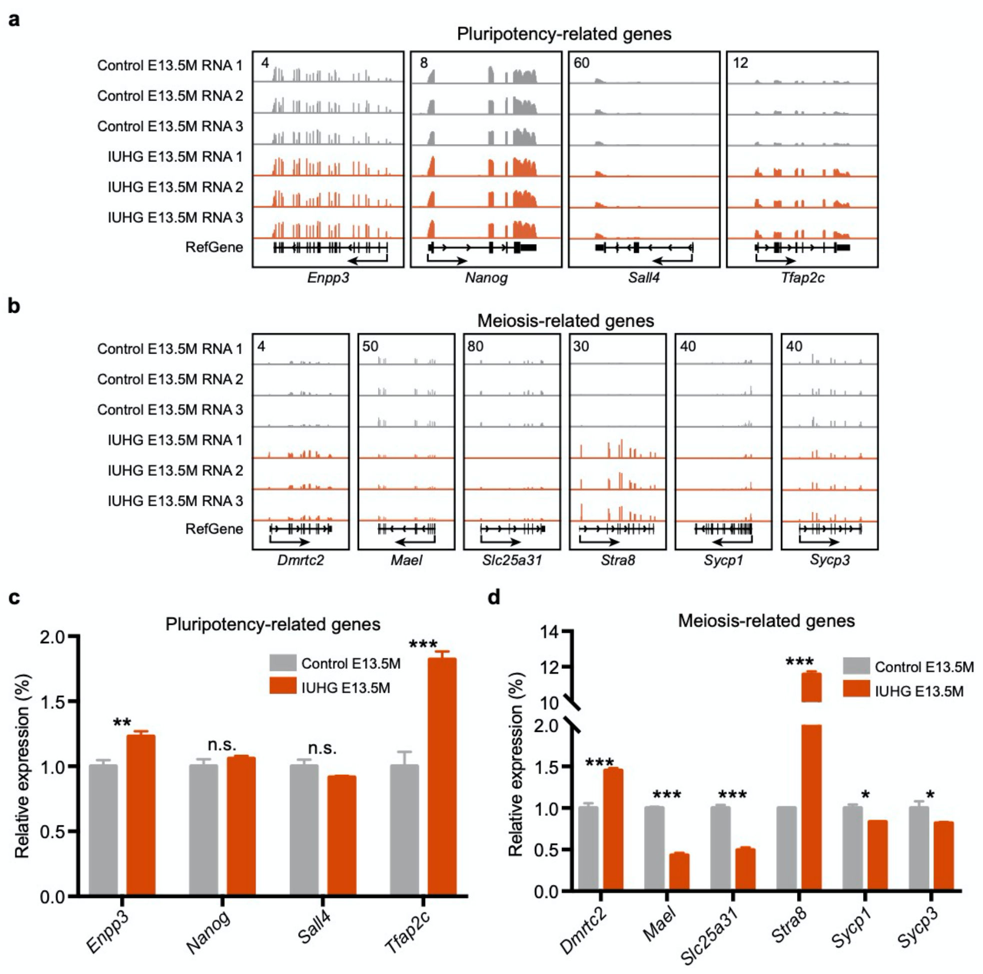

Supplementary information, Fig. S8: The expression changes of representative genes in IUHG and control groups at E13.5 male PGCs.

(**a**, **b**) Genome browser view showing the expression of pluripotency-related genes (*Enpp3*, *Nanog*, *Sall4*, and *Tfap2c*) (**a**) and meiosis-related genes (*Dmrtc2*, *Mael*, *Slc25a31*, *Stra8*, *Sycp1*, and *Sycp3*) (**b**) in control and IUHG group male PGCs at E13.5. Three biological replicates per group. (**c**, **d**) qPCR validation of pluripotency-related genes (*Enpp3*, *Nanog*, *Sall4*, and *Tfap2c*) (**c**) and meiosis-related genes (*Dmrtc2*, *Mael*, *Slc25a31*, *Stra8*, *Sycp1*, and *Sycp3*) (**d**) in IUHG E13.5 male PGCs compared with control groups. All error bars represent the mean ± SD of three biological replicates, each derived from PGCs collected from 6 - 10 pairs of gonads per group. Significance was calculated using an unpaired two-sided Student's *t*-test (**c**, **d**); **P* < 0.05, ***P* < 0.01, ****P* < 0.001, n.s.: not significant.

Supplementary information, Fig. S9

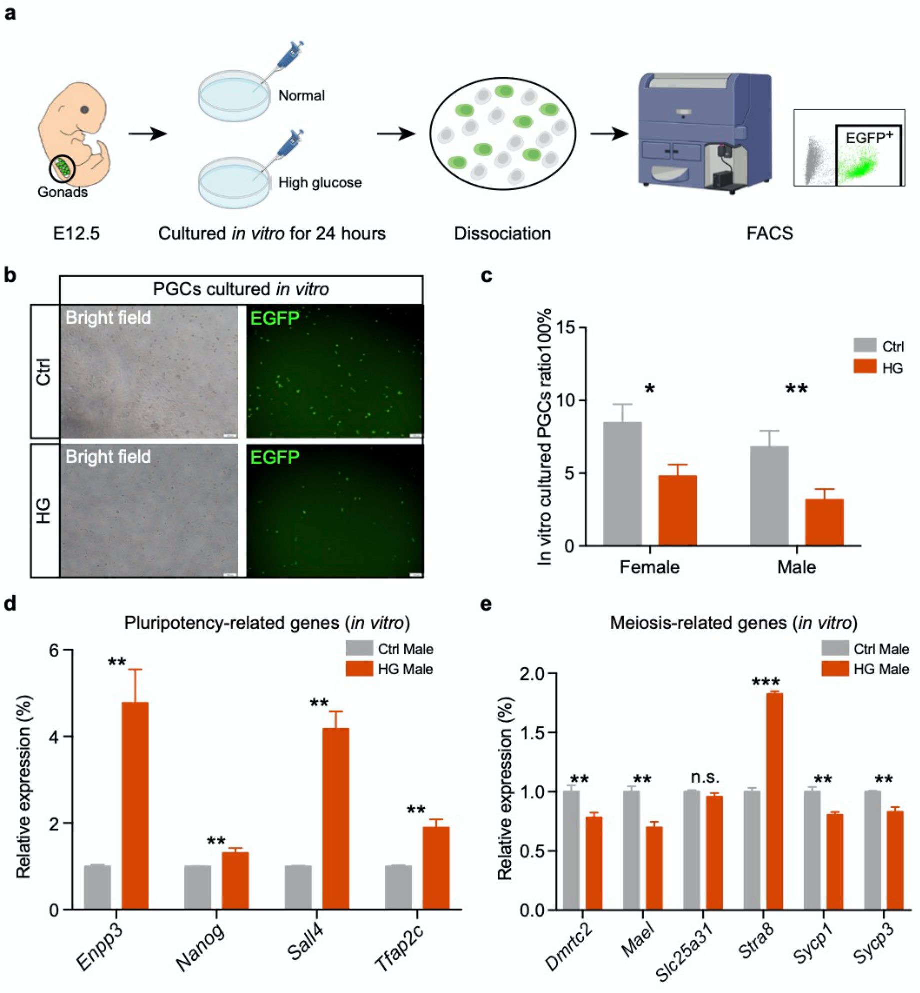

Supplementary information, Fig. S9: *In vitro* exploration of high glucose on PGCs. (**a**) Schematic diagram showing *in vitro* high glucose treatment of E12.5 PGCs for 24 hours. (**b**) E12.5 male PGCs were cultured *in vitro* for 24 hours, scale bars: 100 μm. (**c**) Quantification of average PGC cell numbers sorted by flow cytometry between the control and HG groups cultured *in vitro*. Each group consisted of 6 -10 embryos. (**d**, **e**) Expression of pluripotency-related (**d**) and meiosis-related (**e**) genes in *in vitro* cultured male PGCs by qPCR. All error bars represent the mean ± SD of three biological replicates, each derived from PGCs collected from 6 - 10 pairs of gonads per group. Significance was calculated using an unpaired two-sided Student's *t*-test (**c**, **d**, **e**); $**P < 0.01$, $***P < 0.001$, n.s.: not significant.

Supplementary information, Fig. S10

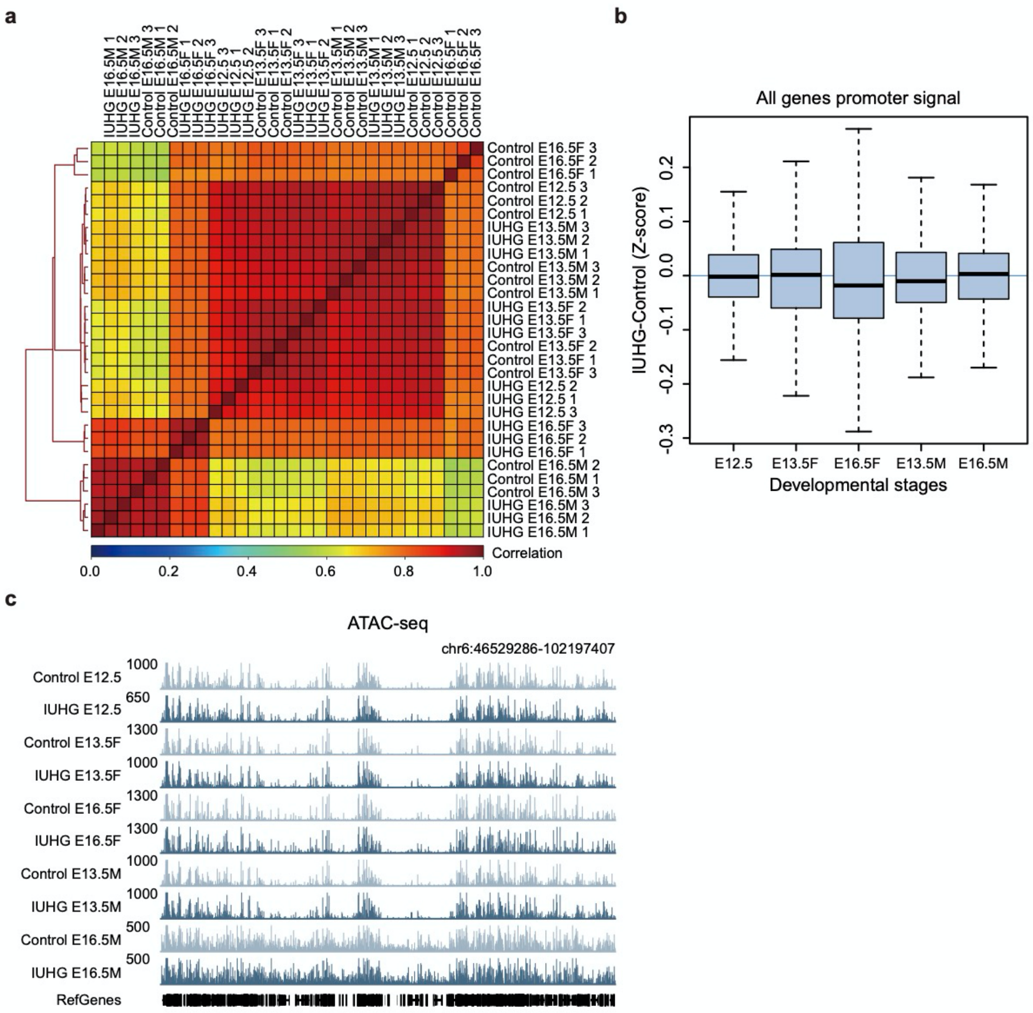

Supplementary information, Fig. S10: Chromatin accessibility datasets during PGC development from E12.5 to E16.5.

(**a**) Heatmap showing the Pearson correlation of chromatin accessibility profiles across developmental stages in control and IUHG groups. (**b**) Boxplot depicting the z-score of promoter accessibility signals for all genes across different developmental stages between control and IUHG groups. (**c**) A snapshot of representative ATAC-seq tracks for chromatin accessibility at a genomic locus (chr6:46,529,286-102,197,407) across different developmental stages from the control and IUHG groups. Three biological replicates were merged for representation.

Supplementary information, Fig. S11

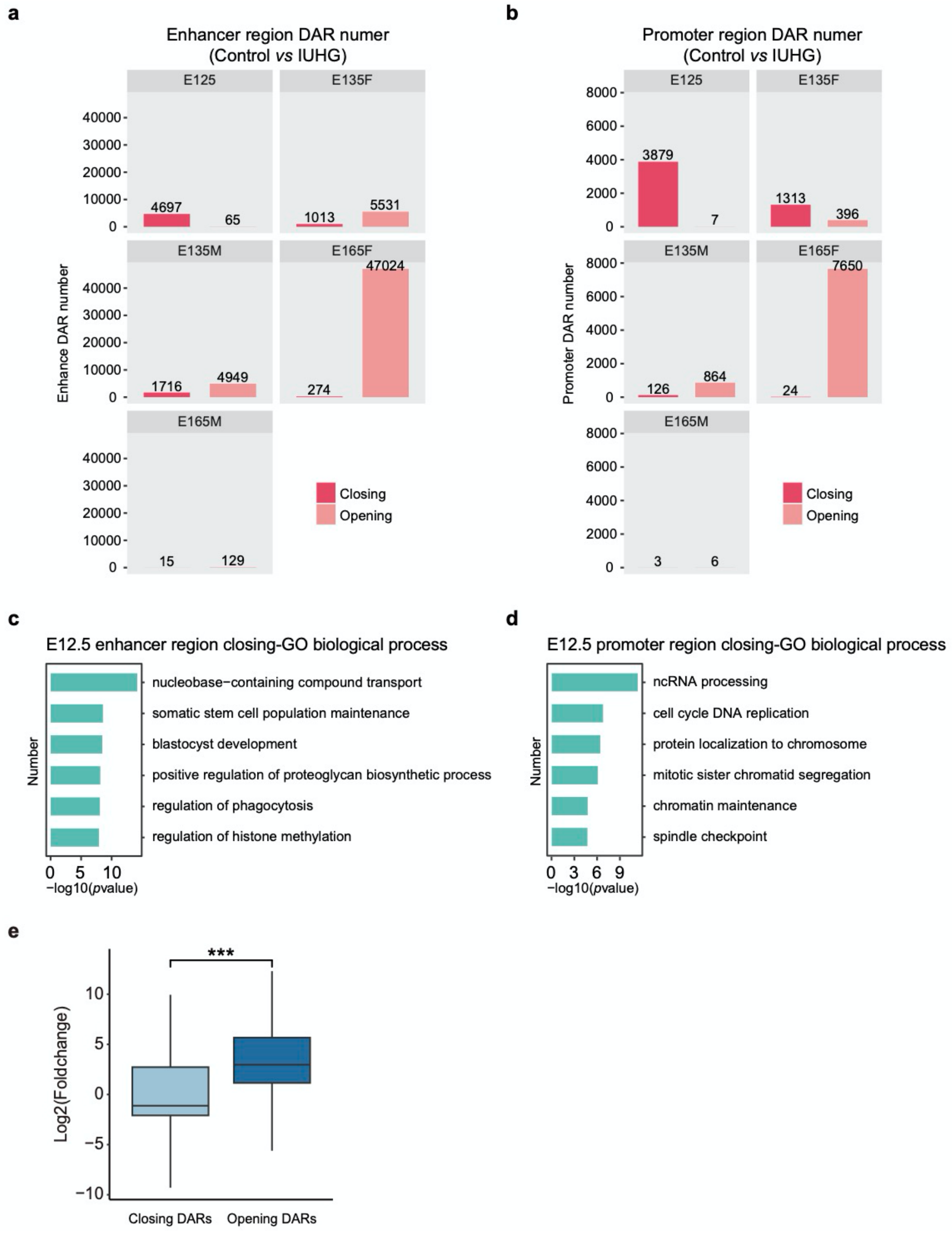

Supplementary information, Fig. S11: Chromatin accessibility dynamics during PGC development from E12.5 to E16.5.

(**a**, **b**) Barplot summarizing the number of differently accessible regions (DARs) identified at each developmental stage at the enhancer (**a**) and promoter (**b**) regions. The DARs are further divided into closing (dark pink) and opening (light pink) regions. (**c**, **d**) GREAT analysis of closing DAR in enhancer (**c**) and promoter (**d**) region at E12.5. (**e**) Gene expression of genes closest to the closing DAR and the opening DAR. Significance was calculated using a Wilcox.test; ***$P < 0.001$.

Supplementary information, Fig. S12

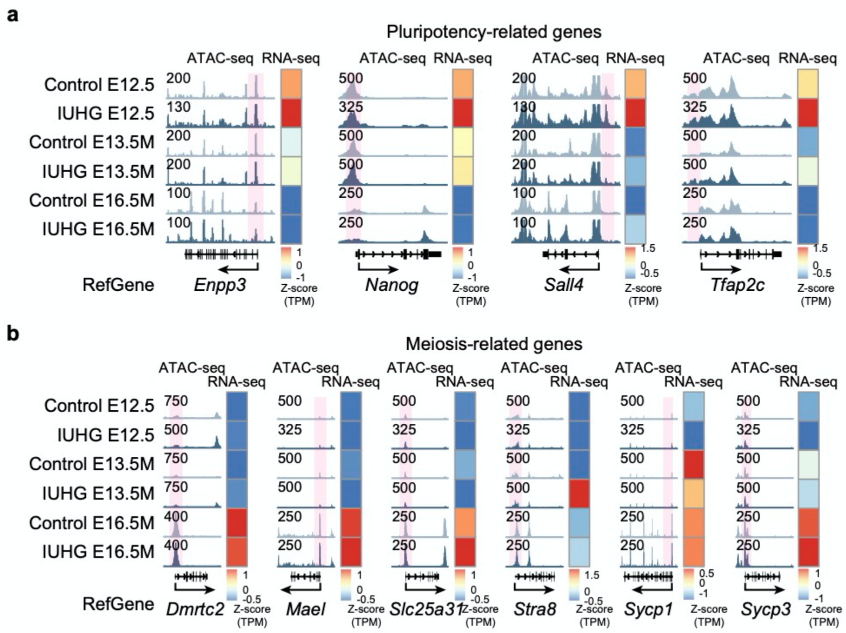

Supplementary information, Fig. S12: Chromatin accessibility and expression changes in pluripotency- and meiosis-related genes in control and IUHG male PGCs.

(**a**) Genome browser view showing the ATAC signal enrichment at core pluripotency TF binding sites around the *Enpp3*, *Nanog*, *Sall4*, and *Tfap2c* loci in male PGCs at E12.5-E13.5-E16.5 between IUHG and control groups. Heatmaps show the expression of those genes. (**b**) Genome browser view showing the ATAC signal enrichment at core meiosis TF binding sites around the *Dmrtc2*, *Mael*, *Slc25a31*, *Stra8*, *Sycp1*, and *Sycp3* loci in male PGCs at E12.5-E13.5-E16.5 between IUHG and control groups. Heatmaps show the expression of those genes.

Supplementary information, Fig. S13

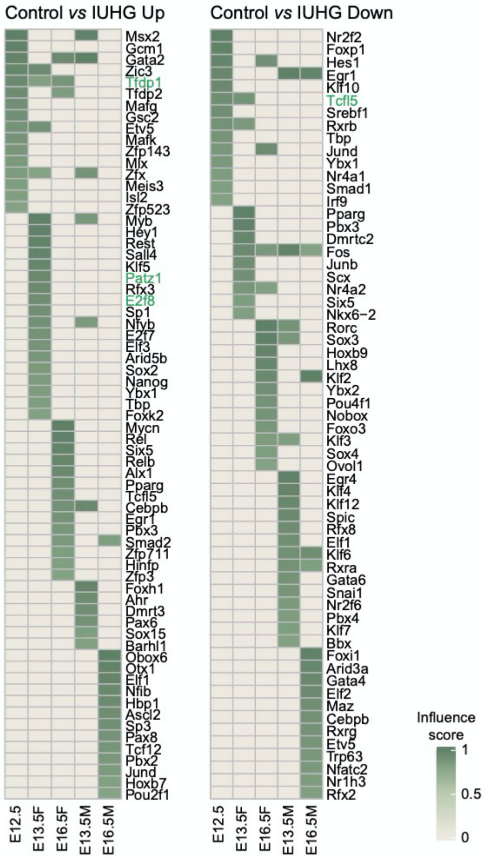

Supplementary information, Fig. S13: Prediction of driver TFs under hyperglycemia condition.

RNA-seq and ATAC-seq linkage analysis to analyze the key transcription factors that drive the state transition between the control and the IUHG group. Genes consistent with the prediction results in **Figure 4g** and **i** are highlighted in green.

Supplementary information, Fig. S14

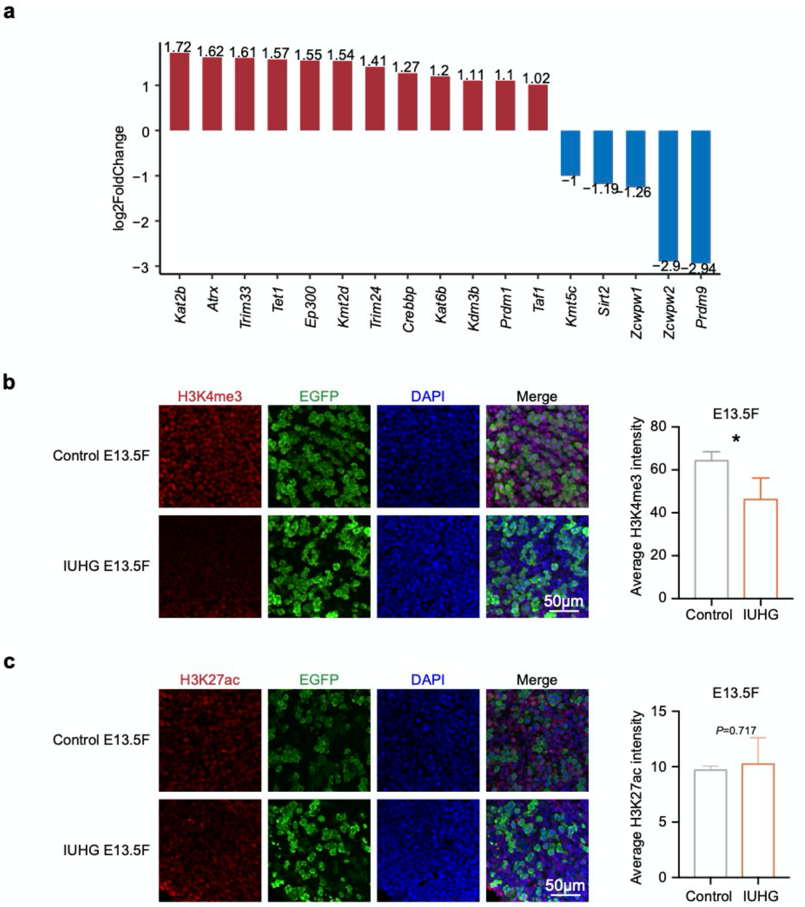

Supplementary information, Fig. S14: Altered histone modifications in E13.5 female germ cells under intrauterine hyperglycemia

(**a**) Bar plots show the top differentially expressed epigenetic regulators ($\log_2$ fold change > |1|) in E13.5 female germ cells between IUHG and control groups based on RNA-seq. (**b**) Immunofluorescence staining and quantification of H3K4me3 (red), a histone mark associated with active chromatin, in EGFP-positive E13.5 female germ cells (green) under control and IUHG conditions. Nuclei were counterstained with DAPI (blue). A significant reduction in H3K4me3 intensity was observed in the IUHG group (*n* = 3 slides, *n* = 3 mice per group). Scale bar: 50 μm. (**c**) Immunofluorescence staining and quantification of H3K27ac (red), another active histone modification, also showed no significant difference between groups (*n* = 3 slides, *n* = 3 mice per group). EGFP marks germ cells; DAPI stains nuclei. Scale bars: 50 μm. All data are presented as mean ± SD. Significance was calculated using an unpaired two-sided Student's *t*-test (**b**, **c**); $*P < 0.05$.

Supplementary information, Fig. S15

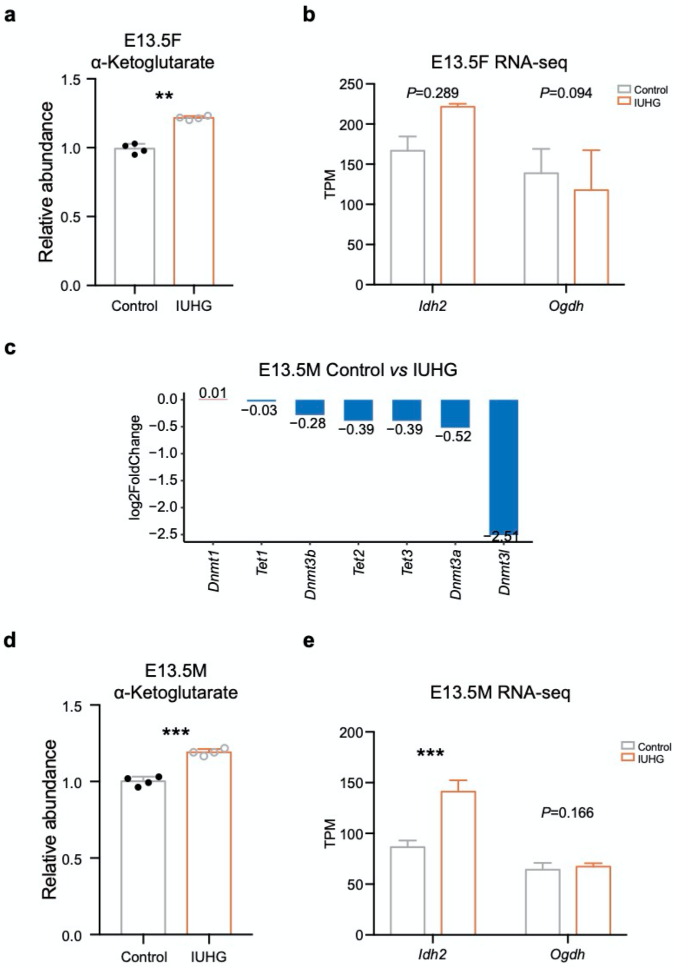

Supplementary information, Fig. S15: Alterations in α-ketoglutarate levels and TCA cycle-related gene expression in E13.5 gonads under intrauterine hyperglycemia.

(**a**) Quantification of α-ketoglutarate (α-KG) levels using a biochemical assay reveals a significant elevation in the IUHG E13.5 female group (n = 4 biological replicates per group, each comprising 20 - 30 pairs of gonads). (**b**) RNA-seq analysis of TCA cycle-related genes *Idh2* and *Ogdh* in E13.5 female gonads shows a non-significant trend toward increased *Idh2* and decreased *Ogdh* expression in the IUHG group compared to controls (n = 3 per group). (**c**) RNA-seq analysis showing the $\log_2$ fold change in expression of genes encoding DNA methylation-related genes in E13.5 male PGCs. (**d**) Quantification of α-KG levels using a biochemical assay reveals a significant elevation

in the IUHG E13.5 male group (n = 4 biological replicates per group, each comprising 20 - 30 pairs of gonadal ridges). (**e**) RNA-seq analysis of TCA cycle-related genes *Idh2* and *Ogdh* at E13.5 male gonads in the IUHG group compared to controls (n = 3 per group). All data are presented as mean ± SD. Student's t-test is used in **a** and **d**; Wald's test calculated by DEseq2 is used in **b** and **e**; $^{**}P < 0.01$; $^{***}P < 0.001$.

Supplementary information, Fig. S16

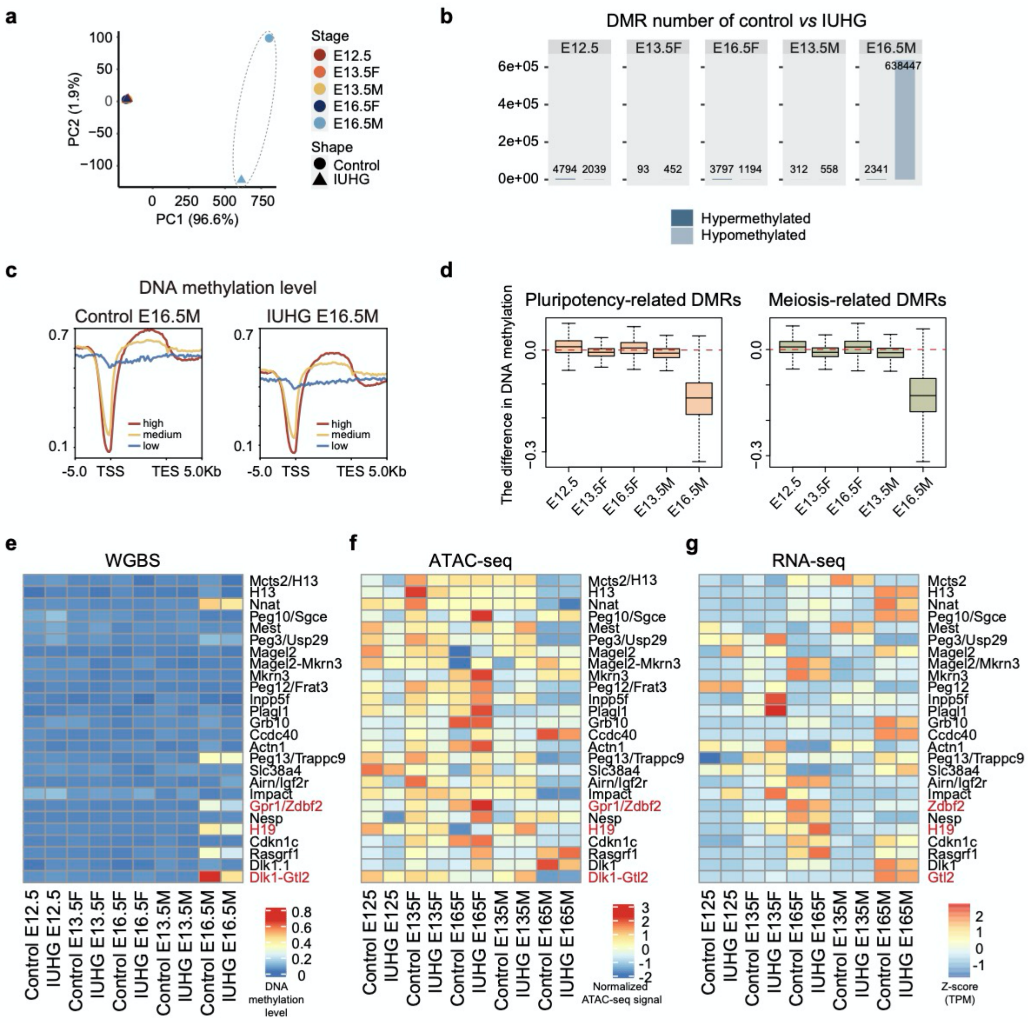

Supplementary information, Fig. S16: The dynamics of DNA methylation during PGC development between control and IUHG groups.

(**a**) PCA analysis shows average methylation levels in the developmental trajectory of PGCs from E12.5 to E16.5. The plot highlights differences in DNA methylation profiles between the control (circle) and IUHG (triangle) groups. Each group includes three biological replicates. (**b**) Barplots summarize the number of differentially methylated regions (DMRs) identified at each developmental stage. The DMRs are divided into hypermethylated (dark blue) and hypomethylated (light blue) regions. (**c**) DNA methylation levels across the transcription start site (TSS) and transcription end site (TES) regions in E16.5 male PGCs from the control (left) and IUHG (right) groups. (**d**) Boxplots illustrate the differences in DNA methylation levels at pluripotency- and

meiosis-related DMRs between the control and IUHG groups. (**e**) Heatmaps show DNA methylation levels at ICRs across different developmental stages (E12.5, E13.5, and E16.5) between control and IUHG groups. (**f**) Heatmaps illustrate chromatin accessibility at the same imprinted loci. (**g**) Heatmaps display gene expression changes of the imprinted genes.

Captions Tables for S1 to S7

Table S1.

Pluripotency-related genes TPM values.

Table S2.

Meiosis-related genes TPM values.

Table S3.

Predicted regulatory TFs TPM values.

Table S4.

Samples used for RNA-seq library construction.

Table S5.

Primers used for qPCR assays.

Table S6.

Samples used for ATAC-seq library construction.

Table S7.

Samples used for WGBS library construction.
